# Inferences to estimate consumer’s diet using stable isotopes: Insights from a dynamic mixing model

**DOI:** 10.1371/journal.pone.0263454

**Published:** 2022-02-07

**Authors:** Marine Ballutaud, Morgane Travers-Trolet, Paul Marchal, Stanislas F. Dubois, Carolina Giraldo, Andrew C. Parnell, M. Teresa Nuche-Pascual, Sébastien Lefebvre

**Affiliations:** 1 Univ. Lille, Univ. Littoral Côte d’Opale, CNRS, IRD, UMR 8187 Laboratoire d’Océanologie et de Géosciences, Lille, France; 2 EMH, Centre Atlantique, Ifremer, Nantes, France; 3 Channel and North Sea Fisheries Research Unit, Ifremer, Boulogne-sur-Mer, France; 4 Laboratory of Coastal Benthic Ecology, DYNECO, Ifremer, Plouzané, France; 5 Hamilton Institute, Insight Centre for Data Analytics, Maynooth University, Maynooth, Ireland; MARE – Marine and Environmental Sciences Centre, PORTUGAL

## Abstract

Stable isotope ratios are used to reconstruct animal diet in trophic ecology via mixing models. Several assumptions of stable isotope mixing models are critical, i.e., constant trophic discrimination factor and isotopic equilibrium between the consumer and its diet. The isotopic turnover rate (λ and its counterpart the half-life) affects the dynamics of isotopic incorporation for an organism and the isotopic equilibrium assumption: λ involves a time lag between the real assimilated diet and the diet estimated by mixing models at the individual scale. Current stable isotope mixing model studies consider neither this time lag nor even the dynamics of isotopic ratios in general. We developed a mechanistic framework using a dynamic mixing model (DMM) to assess the contribution of λ to the dynamics of isotopic incorporation and to estimate the bias induced by neglecting the time lag in diet reconstruction in conventional static mixing models (SMMs). The DMM includes isotope dynamics of sources (denoted *δ*_*s*_), λ and frequency of diet-switch (*ω*). The results showed a significant bias generated by the SMM compared to the DMM (up to 50% of differences). This bias can be strongly reduced in SMMs by averaging the isotopic variations of the food sources over a time window equal to twice the isotopic half-life. However, the bias will persist (∼15%) for intermediate values of the *ω*/λ ratio. The inferences generated using a case study highlighted that DMM enhanced estimates of consumer’s diet, and this could avoid misinterpretation in ecosystem functioning, food-web structure analysis and underlying biological processes.

## Introduction

The use of stable isotope ratios as natural recorders in biotic and abiotic molecules has provided strong support for deciphering ecological processes [1]. These recorders provide an “isotopic signature” that is used to trace the origin and fate of elements (C, N, S) within the biosphere and more particularly, to inform on animal migration patterns and feeding strategies for instance [2]. In trophic ecology, stable isotope ratios (expressed in *δ* notation by convention see Table 1 for a list of symbols and abbreviations) of carbon, nitrogen and sulphur (*δ*^13^C, *δ*^15^N and *δ*^34^S, respectively) enable us to reconstruct animal diets, to characterize trophic interactions [3], to estimate niche breadth [4] and food-web structure [5]. Historically, animal diets were reconstructed using stable isotope analysis based on the simple but constrained premise “You are what you eat (plus a few ‰)” (DeNiro and Epstein 1976 in [6]). It means that stable isotope ratios of one consumer (*δ*_*c*_) resemble that of its assimilated diet (*δ*_*d*_) which is composed of several food source signatures (*δ*_*s*_), plus a difference corresponding to the trophic discrimination factor (noted Δ^13^C and Δ^15^N for carbon and nitrogen, respectively) [7]. Based on this premise, stable isotope mixing models are a widely used method to estimate food source proportions in the consumer’s diet, knowing the incorporated *δ*_*d*_ of an individual, or a group of individuals [8]. Stable isotope mixing models were developed based on two strong assumptions: i) the specific predator-prey trophic discrimination factor distribution is known and constant over time, but also its intra-population variation is smaller than the existing difference between the isotopic signatures of the food sources and, ii) the isotopic equilibrium is reached between *δ*_*d*_ and *δ*_*c*_ [9]. On the one hand, the distribution of the trophic discrimination factor has been extensively studied (e.g., Post [10], Healy et al. [11], McCutchan et al. [12], Caut et al. [13]) to limit its impact on the results of mixing models that are extremely sensitive to its value [14, 15]. On the other hand, assuming an isotopic equilibrium is questionable in most cases, and probably does not occur under natural conditions [16–19]. *δ*_*c*_ does not imprint *δ*_*d*_ instantly, but rather after a time lag that depends on the isotopic turnover rate of the tissue considered (λ, [20]) itself in relation to the physiological state of the consumer [21]. λ is the instantaneous rate of isotopic incorporation with 1/λ the average retention time of an element in a tissue, and ln(2)/λ as its half-life (t_1/2_, [22]). To circumvent the time lag issue when using stable isotope mixing models, Phillips et al. [9] recommended adjusting the sampling window according to λ for a space and/or time averaged collection of potential *δ*_*s*_ values incorporated by the consumer. The simple premise then becomes: You are what you eat plus a few ‰ after a time lag. However, an explicit consideration and quantification of the time lag in stable isotope-based tools is most often missing [20].

**Table 1:** List of acronyms and notations used.

**Acronyms**	**Meaning**
DMM	Dynamic mixing model
SMM	Static mixing model
SMM_t_	Instantaneous static mixing model
SMM_Δt_	Integrated static mixing model
*δ*-space	Isotopic space
*p*-space	Diet proportion space
**Notations**	**Definitions (units)**
*δ*_*c*_, *δ*_*d*_, *δ*_*s*_	Stable isotope ratios of consumer *c*, diet *d* and sources *s* respectively (‰)
Δ_*s*(*i*)_	Food source-specific trophic discrimination factors (‰)
λ	Isotopic turnover rate also named isotopic incorporation rate (d^-1^)
*t*_1/2_	Isotopic half-life of tissues (in d)
*ω*	Frequency of diet-switch (d^-1^)
*p*_*s*_	Proportion of food source *s* into diet
${\hat{p}}_{s}$	Estimated proportion of food source *s* into diet
Δ*t*	Time window of isotopic integration (in d)
$\beta_{\frac{\omega }{\lambda }}$	Bias estimation as a function of the ratio *ω*/λ
*S*	Number of food sources (2 in the *in-silico* experiment, noted *a* and *b*)
*T*	Simulation time (set to 500 d here)

The stable isotope ratios of an organism (primary producer or consumer) evolve more or less rapidly over time but are rarely stable. Three main factors are involved in these dynamics: the stable isotope ratios of the resources used, the proportion of these resources ultimately used to produce tissues and finally the rate of isotopic incorporation. For example, environmental fluctuations cause temporal variability in the isotopic values of marine primary producers due to changes in the nutrient availability [23–25]. These dynamics may produce bias both in the interpretation in diet outputs of isotope mixing models for one consumer [26] and in the assessment of trophic levels in a community [27]. Most animals move across certain areas when foraging, and/or face seasonal fluctuations in resource availability and/or experience ontogenetic diet shifts during their lifespan. For example, sperm whales change their foraging behaviour seasonally, which is expressed by seasonal isotope variations due to a change in habitat or prey [28]. Sessile consumers such as the Pacific oyster face seasonal variations in the availability of their food sources [29]. Anadromous species such as Chinook salmon migrate from freshwater to marine ecosystems as juveniles and backwards as adult [30]. Finally, time lag is proportional to λ and its counterpart the isotopic half-life. Recently, literature meta-analyses were conducted to explore some drivers of λ variations, with λ being estimated from mass or time models [17, 31]. These two studies showed that λ scales isometrically with body mass, a relationship previously predicted by Carleton & Martínez del Rio [32], such as λ is inversely proportional to body mass to power the allometric coefficient. Consequently, λ decreases when body mass increases for a single individual over its lifespan and λ is on average lower for larger species. Suboptimal physiological states of consumers and diet quality are also determinants of λ dynamics [21]. The effects of body mass on λ was clearly evidenced when considering either the sole muscle tissues or the whole body, but the relationships were impaired when using plasma, liver or blood tissues [31]. Thus, it is expected that time lag increases with body mass and time lag can be estimated roughly over an animal’s lifespan from body mass for muscle tissues and the whole body.

Most animals move across certain areas when foraging and/or over the course of their growth. To date, isotopic studies that considered λ explicitly in their inferences have investigated individuals migrating between isotopically distinct habitats [33, 34] and individuals exploiting seasonally available resources [35]. The combination of ontogenetic shifts, suboptimal physiological states of consumers, changes in availability and/or quality of prey resources [21], and habitat use impact *δ*_*c*_, highlighting λ as a key parameter for the integration time of the incorporated *δ*_*s*_, to track animal movements and niche shifting [30, 36, 37]. An isotopic clock based on the comparative analysis of different tissues with tissue-specific isotopic turnover rates can then be constructed to estimate the time elapsed since the diet shift, provided that diet shift was not gradual but occurred at a specific time point [34, 38]. When shifts in diet are frequent, a simple isotopic clock cannot be applied. Given that the seasonal *δ*_*s*_ variations are propagated to higher trophic levels [39, 40] and that consumer foraging behaviours are dynamic over time and/or space [41], it is indispensable to develop a mechanistic framework to study the impact of a changing diet on *δ*_*c*_. Following this path, Yeakel et al. [41] showed that the variance in isotopic niche—i.e., the distribution of isotopic values of the consumer’s sources—for one consumer is systematically high, when its *δ*_*c*_ is in transition phase during a diet-switch. A first attempt of a dynamic mixing model (DMM) integrating λ explicitly was carried out to unravel the diet of a marine suspension-feeder [29]. This study considered both the seasonal variability in *δ*_*s*_ and the variations in λ of the consumer using a bioenergetic model. However, the gain in accuracy brought by this new dynamic approach relative to conventional inferential methods (i.e., the static mixing model, SMM) has not been evaluated. The dynamics of both *δ*_*d*_ and *δ*_*c*_ are not explicitly captured in SMMs. Therefore, estimates of diet contributions using SMM may be biased, regardless of their sensitivity to the trophic discrimination factor used [15], and this bias should be quantified. In a dynamic context (changing habitat, seasonal source variations or prey switching), the explicit consideration of a dynamic λ would allow isotopic ecologists to decipher the dynamics of a consumer’s diet—and thus the dynamics of trophic interactions, which are paramount to understand trophic relationships, food-web structure and ultimately ecosystem functioning [42].

Many efforts have been dedicated to enhancing stable isotope mixing models—concentration dependency [43], combining sources [44], uncertainty of estimates [45], Bayesian framework [8]—but never in a dynamic context and hence, λ dynamics have been neglected so far. In this study, we propose to build and evaluate a dynamic mixing model (DMM), by combining a mechanistic approach (i.e., with stable isotope dynamics) and an inferential approach (i.e., with diet back-calculation). The specific aims of this paper were i) to implement λ into a SMM (i.e., providing a DMM); ii) to use mechanistic simulations of *δ*_*c*_ (that account for different components of temporal variability) in order to estimate food source proportions and the bias occurring when a SMM is applied rather than a DMM; iii) to illustrate the differences in DMM over SMM when estimating food source proportions using case-study data in an inferential framework and; iv) to provide recommendations when using DMMs in isotopic approaches.

## Materials and methods

First, a dynamic mixing model (DMM) was set up and then used into an *in-silico* experiment to depict how the dynamics of several forcing variables impact i) the stable isotope ratios of one consumer (*δ*_*c*_) and, ii) the inferences on consumer’s diet (as proportion of food sources $\hat{p}$). The impacts on the inference were quantified by estimating the bias among different static mixing model (SMM) methods in relation to DMM. Second, a case study based on an existing dataset was used to further highlight our findings.

### Dynamic mixing model framework

For a given element (i.e., C, N, S), the isotopic incorporation dynamics of one consumer over time (*δ*_*c*_(t) in ‰) switching to a new constant diet with a constant isotopic turnover rate (λ in d^-1^), are classically apprehended by the first-order kinetic one-compartment time model [22, 46] which is written as:

$$\begin{array}{c} \delta_{c}\left(t\right)=\delta_{c}\left(\infty \right)+(\delta_{c}\left(0\right)-\delta_{c}\left(\infty \right))\text{exp}(-\lambda t) \end{array}$$ (1)

where *δ*_*c*_(0) is the initial *δ*_*c*_ value before the diet switch (at *t* = 0), and *δ*_*c*_(∞) is the *δ*_*c*_ value at the asymptote (at *t* → ∞), i.e., when the consumer reaches the isotopic equilibrium with its new diet. In a dynamic framework, the evolution of *δ*_*c*_ over time is given by the derivative form of Eq (1):

$$\begin{array}{c} \frac{d\delta_{c}}{dt}=\lambda (\delta_{c}\left(\infty \right)-\delta_{c}) \end{array}$$ (2)

Actually *δ*_*c*_(∞) is the sum of two components: the isotopic value of the diet (*δ*_*d*_(t) in ‰) and the associated trophic discrimination factors (Δ_*s*(*i*)_ in ‰). *δ*_*c*_(∞) is variable since it depends on the dynamic diet *δ*_*d*_(*t*), which corresponds to the dynamic mixture of the signature of *S* incorporated food sources *i* noted *δ*_*s*(*i*)_(*t*) over time.

A classical approach is to correct the *δ*_*c*_ (or equivalently the sources *δ*_*s*_) by the value of Δ. Recently, using combined food source-specific trophic discrimination factors rather than a single and constant value for a given consumer has been shown to improve the results of trophic studies of omnivores [47]. Then, *δ*_*d*_(*t*) is determined by a standard linear mixing model [48] which is the weighted sum of *δ*_*s*(*i*)_(*t*), each corrected of their specific Δ_*s*(*i*)_, and their proportions *p*_*s*(*i*)_(*t*) to the mixture over time:

$$\begin{array}{c} \delta_{d}\left(t\right)=\sum_{i=1}^{S}p_{s(i)}\left(t\right)(\delta_{s(i)}\left(t\right)+\Delta_{s(i)}) \end{array}$$ (3)

The linear mixing model Eq (3) is classically used as a static case (i.e., *δ*_*d*_ is constant and independent of time) under the assumption of isotopic equilibrium, and this configuration is hereafter named the static mixing model (SMM). The sum of *p*_*s*(*i*)_(*t*) equals to 1 at each time step, and all sources are assumed to be identified giving: $\sum_{i=1}^{S}p_{s(i)}\left(t\right)=1$. The dynamic mixing model (DMM) merges the time model Eq (2) and the linear mixing model Eq (3) as a first-order ordinary differential equation:

$$\begin{array}{c} \frac{d\delta_{c}}{dt}=\lambda \left(t\right)((\sum_{i=1}^{S}p_{s(i)}\left(t\right)(\delta_{s(i)}\left(t\right)+\Delta_{s(i)}))-\delta_{c}) \end{array}$$ (4)

where λ(*t*), *δ*_*s*(*i*)_(*t*) and *p*_*s*(*i*)_(*t*) vary over time but Δ_*s*(*i*)_ is constant (see Table 1 for notations and units). In the particular case of a constant λ over time, an analytical solution for Eq (4) exists and has been detailed by Yeakel et al. [41], and is composed of two terms: a first one diluting the initial value of the consumer and a second one integrating the value of the diet. However, in a full dynamic framework when λ varies over time, there is no analytical solution for Eq (4) and it is resolved numerically in this study.

#### Computing procedure of the dynamic mixing model

The dynamic mixing model (DMM, Eq (4)) is an ordinary differential equation expressing the isotopic value of consumer over time, and depending on forcing variables as the isotopic value of its diet and the turnover rate. Eq (4) does not have an analytical solution in its fully dynamic version. However, such an ordinary differential equation can be solved numerically using the package deSolve [49]. In order to run the numerical solver function (named lsoda in deSolve), the specification of forcing variables and initial state are necessary. The initial state was the first isotopic value of consumer. As the numerical solver uses the integration of the Runge-Kutta family, the forcing variables must be continuous over time and not only defined for discrete sampling dates. Therefore the forcing variables are interpolated linearly using the approxfun() function. The DMM is coded in R language (version 4.1.2) and the entire code to reproduce all figures and to use the model is provided on GitHub (available at https://github.com/mballutaud/isotroph/tree/master/dmm).

### *In-silico* experiment and bias estimates in inferences

Once the dynamic mixing model (DMM) is set up, the aim of the *in-silico* experiment is to evaluate the bias generated on inferences when using a static mixing model (SMM) compared to a DMM for a given forcing *δ*_*d*_(*t*). The components of the variability in isotopic values of consumer *δ*_*c*_(*t*) (i.e., isotopic values of food sources *δ*_*s*(*i*)_(*t*), source proportions to the diet *p*_*s*(*i*)_(*t*) and isotopic turnover rate λ(*t*)) are explicitly incorporated into the DMM to produce simulations accounting for their combined effects in a mechanistic approach.

#### Components of variability

Three temporal components of variability were identified and factorized (i.e., *δ*_*s*(*i*)_(*t*), *p*_*s*(*i*)_(*t*) and λ(*t*)), and implemented in the DMM Eq (4) as forcing variables. The trophic discrimination factor could also be a source of variability. However, for the sake of simplicity and ease of interpretation, we consider trophic discrimination factor to be constant in our *in-silico* experiment. We apply our modelling framework to atomic element C, for which the trophic discrimination factors were set to 1 ‰ for both food sources [6]. The *S* dynamics of *δ*_*s*(*i*)_(*t*) are described with independent and random trajectories, each of them following a Brownian motion:

$$\begin{array}{c} \delta_{s(i)}\left(t+1\right)=\delta_{s(i)}\left(t\right)+V\left(t\right) \end{array}$$ (5)

With

$$\begin{array}{c} V\left(t\right)∼N\left(0,\sigma^{2}\right) \end{array}$$ (6)

*δ*_*s*(*i*)_(*t* + 1) depends on its value at the previous time step (*t*) plus a random value (*V*(*t*)) that follows a centred normal law Eq (6) with a variance *σ*^2^ set here to 0.2. For two sources (*a* and *b* hereafter, with their signature over time noted respectively *δ*_*s*(*a*)_(*t*) and *δ*_*s*(*b*)_(*t*)), *δ*_*s*(*a*)_(0) was set to 0 ‰ and *δ*_*s*(*b*)_(0) to 10 ‰. Constant isotopic values of food sources {*δ*_*s*(*a*)_(*t*); *δ*_*s*(*b*)_(*t*)} can be modelled by setting the variance to zero (i.e., *σ*^2^ = 0) so *V*(*t*) is null in Eqs (5) and (6). The selection of Brownian motion aimed at simulating trajectories that typically mimic time variations of *δ*_*s*_ in natural conditions. Some 150 trajectories were simulated for each *δ*_*s*(*a*)_(*t*) and *δ*_*s*(*b*)_(*t*) independently. When using stable isotopes mixing models, *δ*_*s*(*a*)_(*t*) and *δ*_*s*(*b*)_(*t*) must have distinct values to infer diet contributions correctly [9, 50]. To prevent situations where *δ*_*s*(*a*)_(*t*) and *δ*_*s*(*b*)_(*t*) would be confounded, or where the time trajectories of *δ*_*s*(*a*)_(*t*) and *δ*_*s*(*b*)_(*t*) would cross each other, a filter was built: ∣*δ*_*s*(*a*)_(*t*) − *δ*_*s*(*b*)_(*t*)∣ ≥ 2 ‰. This filter allowed selecting randomly 100 valid trajectories among the 150 available trajectories, so to impose a minimum difference of 2 ‰ between *δ*_*s*(*a*)_(*t*) and *δ*_*s*(*b*)_(*t*) values for each *t* during the simulated period, noted *T*. For simplicity, *p*_*s*(*i*)_(*t*) was simulated as a binary variable, switching between 0 and 1 at a frequency *ω* (referred to as frequency of diet-switch) that impacts *δ*_*d*_(*t*). In natural conditions, it means that consumers switch completely from one prey to another, or that the prey are mutually exclusive in foraging area. In our *in-silico* experiment, *ω* alternately toggles incorporation of *δ*_*s*(*a*)_(*t*) and *δ*_*s*(*b*)_(*t*) in *δ*_*d*_(*t*). The alternation between *δ*_*s*(*a*)_(*t*) and *δ*_*s*(*b*)_(*t*) is accomplished through the two distinct source proportions *p*_*s*(*a*)_(*t*) and *p*_*s*(*b*)_(*t*) with *p*_*s*(*b*)_(*t*) = 1 − *p*_*s*(*a*)_(*t*). Eq (7) produces oscillated values which are limited by the round function in the R language (included in Base R package version 4.1.2) to return a binary value for *p*_*s*(*a*)_(*t*). Thus, *ω* allows a binary changeover from *p*_*s*(*a*)_(*t*) = 1 to *p*_*s*(*a*)_(*t*) = 0 (Eq (7)), resulting in the incorporation of *δ*_*s*(*a*)_(*t*) or *δ*_*s*(*b*)_(*t*) in *δ*_*d*_(*t*) (Eq (8)).

$$\begin{array}{c} if\;\text{sin}\left(\pi \omega t\right)>0\{\begin{array}{cc} then & p_{s(a)}\left(t\right)=1\\ else & p_{s(a)}\left(t\right)=0 \end{array} \end{array}$$ (7)

$$\begin{array}{c} if\;p_{s(a)}\left(t\right)=1\{\begin{array}{cc} then & \delta_{d}\left(t\right)=p_{s(a)}\left(t\right)(\delta_{s(a)}\left(t\right)+\Delta_{s(a)})\\ else & \delta_{d}\left(t\right)=(1-p_{s(a)}\left(t\right))(\delta_{s(b)}\left(t\right)+\Delta_{s(b)}) \end{array} \end{array}$$ (8)

The last component of variability is λ(*t*). In our *in-silico* experiment, λ is either considered constant and set to different values—between 2.10^−3^ and 2.10^−1^ d^-1^ based on the range of values observed in Thomas & Crowther [31]—or λ is considered dynamic and decreasing through time. The dynamics of λ(*t*) are mimicked using the following equation:

$$\begin{array}{c} \lambda (t)=\lambda (0)\text{exp}(-\alpha t) \end{array}$$ (9)

λ(*t*) represents an exponential decay over time, starting at λ(0) = 2.10^−1^ d^-1^ and with *α* set to 0.01 d^-1^ in order to cover the range of realistic λ values for the time period *T* and to approach 1.10^−3^ d^-1^ at the end of the period. For a small-growing animal, λ decreases with increasing of body mass [51]. Since λ equals the sum of catabolic turnover and mass-specific growth rate [52], then it is realistic to model the dynamics of λ as a decreasing exponential curve over time. This is referred to as the ontogenetic λ scenario hereafter, and corresponds to change in λ values due to the growth of an individual, through several distinct life stages such as larvae, juvenile and adult. For the sake of applicability, the DMM Eq (4) is adapted to *δ*^13^C with two sources *δ*_*s*(*a*)_(*t*) and *δ*_*s*(*b*)_(*t*), each corrected by Δ_*s*(*i*)_ = 1 ‰ in our *in-silico* experiment. The applied DMM then becomes:

$$\begin{array}{c} \frac{d\delta_{c}}{dt}=\lambda \left(t\right)([p_{s(a)}\left(t\right)(\delta_{s(a)}\left(t\right)+\Delta_{s(a)})+(1-p_{s(a)}\left(t\right))(\delta_{s(b)}\left(t\right)+\Delta_{s(b)})]-\delta_{c}) \end{array}$$ (10)

#### Bias in inferences

When the isotopic equilibrium assumption is relaxed, inferences on diet composition using SMM may be biased. DMM in Eq (10) was used to simulate dynamics of *δ*_*c*_(*t*) when {*δ*_*s*(*a*)_; *δ*_*s*(*b*)_}, *ω* and λ are known and vary in a mechanistic framework.

**Fig 1:**
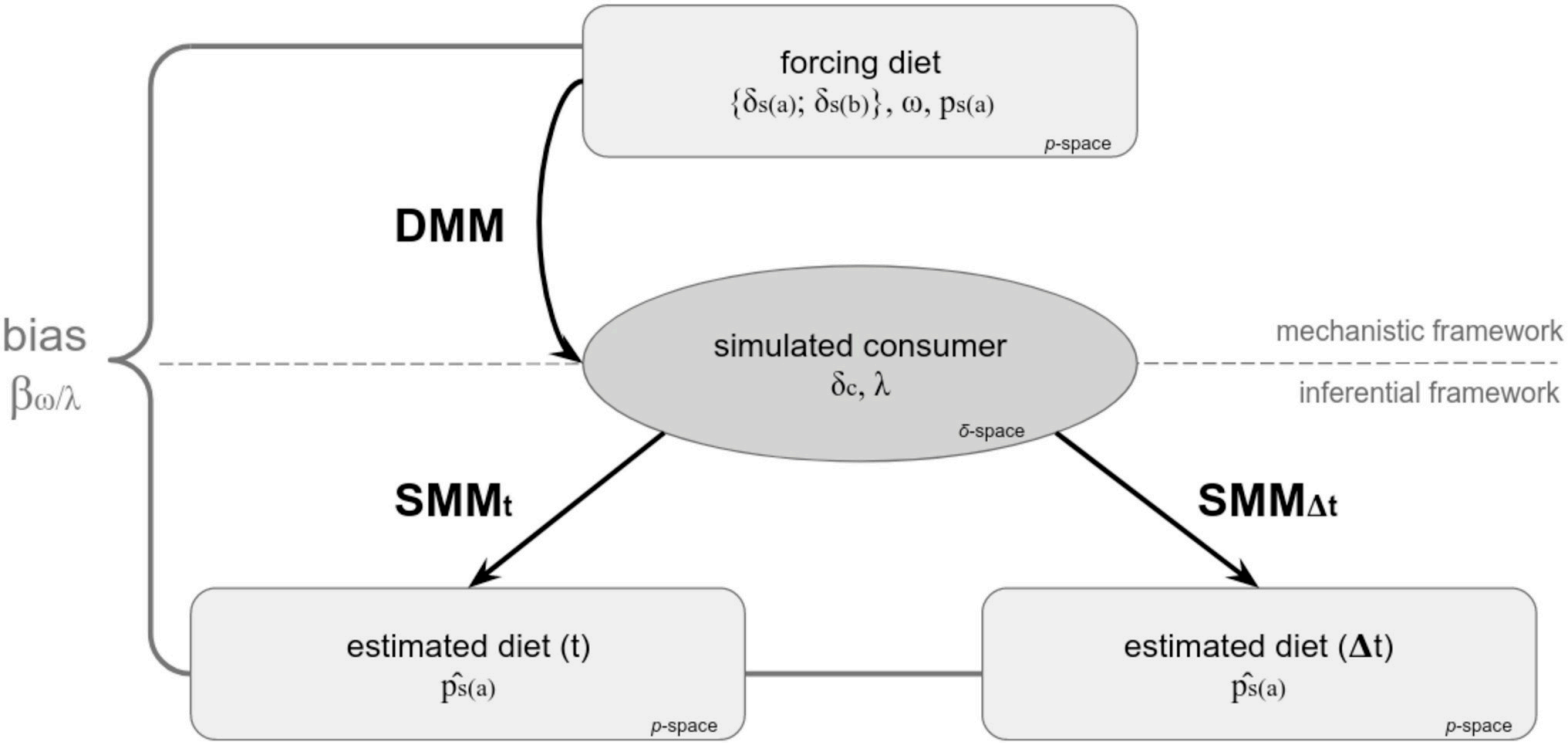
Flowchart representing the procedure for estimating the bias ($\beta_{\frac{\omega }{\lambda }}$) generated by applying inferential methods (SMM_t_, SMM_Δt_). *δ*_*c*_(*t*) is simulated with the mechanistic DMM under different scenarios of forcing diet ({*δ*_*s*(*a*)_; *δ*_*s*(*b*)_}, *ω*) and λ values. In the inferential framework, SMM_t_ and SMM_Δt_ provide respectively instantaneous and integrated estimations of ${\hat{p}}_{s(a)}$. $\beta_{\frac{\omega }{\lambda }}$ is computed by comparing the output of SMMs (i.e., ${\hat{p}}_{s(a)}$) with the initial forced diet (i.e., *p*_*s*(*a*)_ input of the DMM).

From the simulated *δ*_*c*_(*t*) and the forcing {*δ*_*s*(*a*)_(*t*); *δ*_*s*(*b*)_(*t*)}, ${\hat{p}}_{s(a)}\left(t\right)$ is inferred (denoted in comparison to the forced known *p*_*s*(*a*)_(*t*)) through two different SMM methods (Fig 1). The first SMM method allows to estimate ${\hat{p}}_{s(a)}$ at each *t* (Eq (11)) and is named the “instantaneous” method (SMM_t_). Following the recommendation of Phillips et al. [9], the second SMM method is the “integrated” one (SMM_Δt_), which assumes that *δ*_*c*_(*t*) results from the incorporation of *δ*_*d*_(*t*) over a period of time, and requires averaging {*δ*_*s*(*a*)_(*t*); *δ*_*s*(*b*)_(*t*)} over an integration time window (Δ*t*). SMM_Δt_ allows to calculate ${\hat{p}}_{s(a)}$ which is assumed constant over Δ*t*.

$${\hat{p}}_{s(a)}(t)=\left\{\begin{array}{cc} \frac{\left(\delta_{c}(t)-\left(\delta_{s(b)}(t)+\Delta_{s(b)}\right)\right)}{\left(\left(\delta_{s(a)}(t)+\Delta_{s(a)}\right)-\left(\delta_{s(b)}(t)+\Delta_{s(b)}\right)\right)} & {\text{for}\;\text{SMM}}_{\text{t}}\\ \frac{\left(\delta_{c}(t)-\left(\bar{\delta_{s(b)}}(\Delta t)+\Delta_{s(b)}\right)\right)}{\left(\left(\bar{\delta_{s(a)}}(\Delta t)+\Delta_{s(a)}\right)-\left(\bar{\delta_{s(b)}}(\Delta t)+\Delta_{s(b)}\right)\right)} & {\text{for}\;\text{SMM}}_{\Delta \text{t}} \end{array}\right.$$ (11)

Then, the bias ($\beta_{\frac{\omega }{\lambda }}$) of inferring diet using SMMs is computed by comparing the forced diet used as a reference *p*_*s*(*a*)_(*t*) with the inferred one ${\hat{p}}_{s(a)}\left(t\right)$:

$$\begin{array}{c} \beta_{\frac{\omega }{\lambda }}=\{\begin{array}{cc} \frac{\sum_{t=0}^{T}|p_{s(a)}\left(t\right)-{\hat{p}}_{s(a)}\left(t\right)|}{T} & {\text{for}\;\text{SMM}}_{\text{t}}\\ \frac{\sum_{t=\Delta t}^{T}|p_{s(a)}\left(t\right)-{\hat{p}}_{s(a)}\left(t\right)|}{T-\Delta t} & {\text{for}\;\text{SMM}}_{\Delta \text{t}} \end{array} \end{array}$$ (12)

The bias is estimated by summing the absolute value of the difference between *p*_*s*(*a*)_ and ${\hat{p}}_{s(a)}$ at each *t* then dividing by *T* for SMM_t_. As for SMM_Δt_ the bias is also estimated by summing the absolute value of the difference between *p*_*s*(*a*)_ and ${\hat{p}}_{s(a)}$ at each *t* but starting at Δ*t* up to *T* then dividing by *T* − Δ*t* (Eq (12)). ${\hat{p}}_{s(a)}$ and $\beta_{\frac{\omega }{\lambda }}$ are presented in the section of diet proportion space, also named *p*-space.

#### Experimental design

The effects of the three forcing variables *δ*_*s*(*i*)_(*t*), *p*_*s*(*i*)_(*t*) and λ(*t*) were explored in three sets of scenario of increasing complexity, using a limited number of modalities per parameter. The first set of scenarios explores effects of *ω* and λ variabilities and involves combination of *ω* values set to 0.002 d^-1^ (1 switch) or 0.008 d^-1^ (4 switches) with λ constant values set to 2.10^−3^ d^-1^ (low), 2.10^−2^ d^-1^ (intermediate) or 2.10^−1^ d^-1^ (high), while the stable isotope ratios of the source remain constant. Under natural conditions, it means that over a period of 500 d—corresponding to the lifespan of the consumer for example—the consumer performs an ontogenetic shift (*ω* = 0.002 d^-1^) or summer/winter seasonal shifts (*ω* = 0.008 d^-1^). The range of isotopic turnover values will vary between 2.10^−3^ d^-1^ and 2.10^−1^ d^-1^ (retrieved from Thomas & Crowther [31]) using isotopic half-life values of muscle and whole body tissues for endotherms and ectotherms species. A second set of scenarios explores the effect of considering the variability of {*δ*_*s*(*a*)_; *δ*_*s*(*b*)_} using Brownian trajectories in addition to the previous factors. This second set of scenarios corresponds to the seasonal or habitat isotopic variabilities displayed by the prey depending on their own resources availability (e.g., temporal variation of nutrient fluxes for primary producers). The third set of scenarios includes ontogenetic dynamics of λ, on top of all other components of variability. This third scenario represents the real dynamic λ for muscle tissue and growing animal. For each scenario, the dynamics of *δ*_*c*_(*t*) is simulated over *T* (simulation time) of 500 d with every day outputs. The different scenarios provide the simulated value of consumer over time *δ*_*c*_(*t*). The simulated scenarios are presented in the stable isotopic space, a.k.a *δ*-space. In the *p*-space, the comparison between proportions of a forced diet (*p*_*s*(*a*)_) and those estimated in the inferential framework (${\hat{p}}_{s(a)}$) provides some estimates of bias. Bias values were explored for different values of the *ω*/λ ratio and different scenarios of simulated *δ*_*c*_(*t*). In order to explore a potential trend of $\beta_{\frac{\omega }{\lambda }}$, we selected the *ω* values allowing to cover the range of *ω*/λ ratio (i.e., from 0.01 to 4) corresponding to the scenarios of the *in-silico* experiment, for intermediate λ value (i.e., 2.10^−2^ d^-1^). The first value of *ω*/λ was set to 0.01 (different to zero, to run the solver of the DMM) and the next values were set from 0.5 to 4 with a step of 0.5. The λ parameter is set to ensure that Δ*t* does not exceed the length of simulated period (*T*).

### Case study—Inferences on a real data set

Using a real dataset case study, we compared the inferences produced by the dynamic mixing model, and the instantaneous and integrated static mixing models (DMM, SMM_t_ and SMM_Δt_ respectively). To apply the three mixing models, *δ* values of both putative food sources (*δ*_*s*(*i*)_(*t*)) and consumer (*δ*_*c*_(*t*)) are needed at several sampling times (*t*) with further estimates of isotopic turnover rates (λ) for DMM and SMM_Δt_. Then, the contributions of each food sources (*p*_*s*(*i*)_(*t*)) to the diet (*δ*_*d*_(*t*)) can be estimated for each method. This kind of dataset—composed of *δ*_*s*(*i*)_(*t*), *δ*_*c*_(*t*) and λ(*t*) trajectories—is scarce in literature to date. Marín-Leal et al. [29] studied the trophic ecology of the Pacific oyster (*Crassostrea gigas*) in three coastal locations in NW France, over an annual survey. Marine suspension-feeders, such as oysters, typically experience environmental fluctuations and diversity in *δ*_*s*(*i*)_(*t*) leading to variable growth and λ. Marín-Leal et al. [29] suggested to determine the temporal dynamics of *p*_*s*(*i*)_(*t*) to the diets of cultivated oysters from carbon and nitrogen isotopic values (*δ*^13^C and *δ*^15^N), by i) estimating λ(*t*) with bioenergetic modelling (i.e., an estimation of the turnover rate of the whole oyster tissues through a dynamic energy budget (DEB) model), ii) identifying four potential food sources (and hence four *δ*_*s*(*i*)_(*t*) time series) in oyster’s diet, and iii) using two scenarios of trophic discrimination factor for *δ*^13^C and *δ*^15^N. Within the Marín-Leal et al. [29] dataset providing different locations, sampling times and food sources, we selected a subset of them according to the following criteria: i) distinct isotopic values of two main food sources and ii) values of λ over time. The selected dataset includes *δ*_*s*(*i*)_(*t*) values of marine suspended particulate organic matter (PhyOM) and microphytobenthos (MPB) (from Fig 2a in [29]) and *δ*_*c*_(*t*) values for oyster on five dates (bimonthly sampling from May 2004 to January 2005, from Fig 4a in [29]) corresponding to four estimates of λ (estimated between the sampling dates, Fig 5a from [29]) in a same location (BDV-N). In this case study with two food sources and one isotope, the mixing models are fully constrained and provide a unique solution of *p*_*s*(*i*)_(*t*) for each sampling date (*t*). DMM Eq (10), SMM_t_ and SMM_Δt_ Eq (11) were applied to the two main food sources and to the *δ*^13^C values with a trophic discrimination factor value set to 1 ‰ for both sources [10], and to be in the same situation than in our *in-silico* experiment (section entitled *In-silico* experiment and bias estimates in inferences). Eq (11) was applied to estimate diet proportions of microphytobenthos (${\hat{p}}_{s(MPB)}\left(t\right)$) instantaneously via SMM_t_ at each sampling date. For integrating source signatures over Δ*t*, and therefore estimate ${\hat{p}}_{s(MPB)}\left(\Delta t\right)$ using SMM_Δt_, linear interpolations between sampled *δ*_*s*(*i*)_(*t*) values were performed. In the fully dynamic case with DMM Eq (10), an inverse method was used to estimate ${\hat{p}}_{s(MPB)}\left(t\right)$. This consists of testing all possible values of ${\hat{p}}_{s(MPB)}\left(t\right)$ (from 0 to 1, with a resolution of 0.01) for each period encompassed between sampling dates and keeping the one which provided the best fit between the simulated *δ*_*c*_(*t*) and the sampled *δ*_*c*_(*t*) (with a tolerance lower than 0.2 ‰). Each time interval was considered independently by re-initializing the initial *δ*_*c*_(*t*) at the beginning of the period. Similarly to SMM_Δt_, the trajectories of *δ*_*s*(*MPB*)_(*t*) and *δ*_*s*(*PhyOM*)_(*t*) were linearly interpolated between sampled dates to provide a dynamic signal to be implemented in the DMM at each t.

## Results

### Simulations in *δ*-space

**Fig 2:**
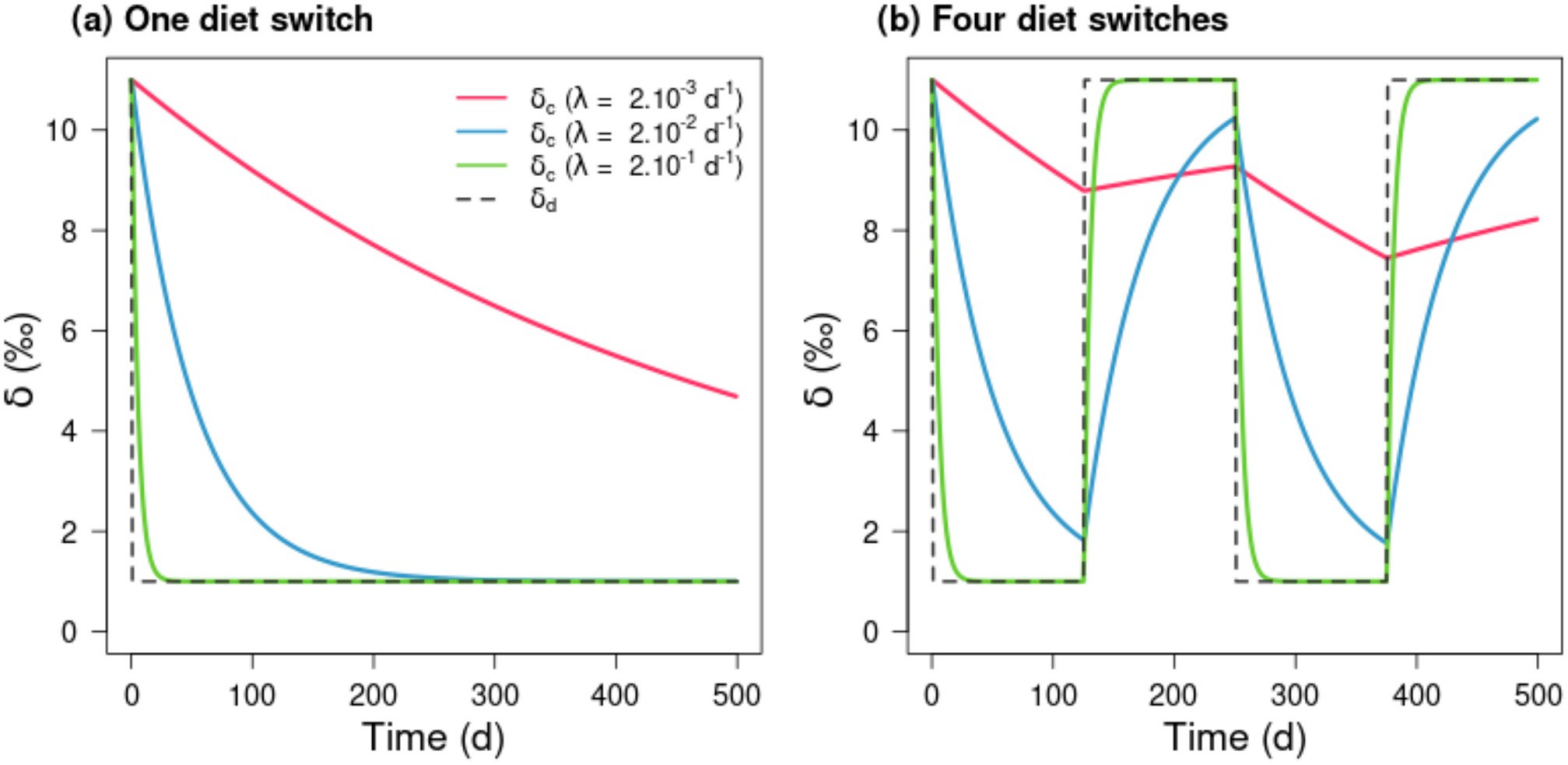
Simulated isotopic values of consumer (*δ*_*c*_(*t*)) during 500 d, for contrasted isotopic turnover rates (λ solid lines) and experiencing a variable diet (*δ*_*d*_(*t*) dashed line) resulting from one (a) or four diet-switches (b) between two food sources (*δ*_*s*(*a*)_ = 0 ‰; *δ*_*s*(*b*)_ = 10 ‰). The λ values were constant and low λ = 2.10^−3^ d^-1^ (red), intermediate λ = 2.10^−2^ d^-1^ (blue), and high λ = 2.10^−1^ d^-1^ (green) corresponding to the range of values of Thomas & Crowther (2015) [31]. The frequencies of diet shift are *ω* = 0.002 d^-1^ (a) and *ω* = 0.008 d^-1^ (b). The ratios *ω*/λ were respectively 1, 0.1 and 0.01 (a) 4, 0.4 and 0.04 (b). Note that for each of the food source, the trophic discrimination factors (Δ_*s*(*i*)_) were set to 1 ‰.

The dynamic mixing model (DMM) was used to mechanistically simulate isotope dynamics of consumer (*δ*_*c*_(*t*)) in *δ*-space. The effects of *ω* on *δ*_*c*_(*t*) for three given and constant values of λ (intermediate, high and low) and of {*δ*_*s*(*a*)_; *δ*_*s*(*b*)_} were analysed first. For one diet switch (*ω* = 0.002 d^-1^, 1 switch at *t* = 0) from *δ*_*d*_(*t*) = 11 ‰ to *δ*_*d*_(*t*) = 1 ‰, the decay rate of *δ*_*c*_(*t*) towards isotopic equilibrium (i.e., when *δ*_*c*_(*t*) tends to *δ*_*d*_(*t*) see Eq (3) with Δ_*s*(*i*)_ = 1 ‰) increased with higher λ values (Fig 2a). Note that when λ is intermediate (blue line) or high (green line), isotopic equilibrium was reached in 250 d or 25 d respectively, while it was not the case with the lowest λ value (time lag larger than 500 d, red line Fig 2a). When diet switches increased (*ω* = 0.008 d^-1^, 4 switches) with alternation of *δ*_*d*_(*t*) between the two food sources (*δ*_*s*(*a*)_ = 0 ‰ and *δ*_*s*(*b*)_ = 10 ‰), the effect of *ω* on *δ*_*c*_(*t*) was drastically amplified when λ decreased and isotopic equilibrium could never be reached for low and intermediate λ values (Fig 2b). With the lowest λ value (red line Fig 2b), *δ*_*c*_(*t*) tended stepwise towards a mid-position between the two food sources corrected by their Δ_*s*(*i*)_ by accumulating the time lags generated by the slow isotopic turnover (Fig 2b). Only for the highest λ value (green line Fig 2b), *δ*_*c*_(*t*) promptly achieved the isotopic values of *δ*_*d*_(*t*) for both *ω* regimes (Fig 2a and 2b).

**Fig 3:**
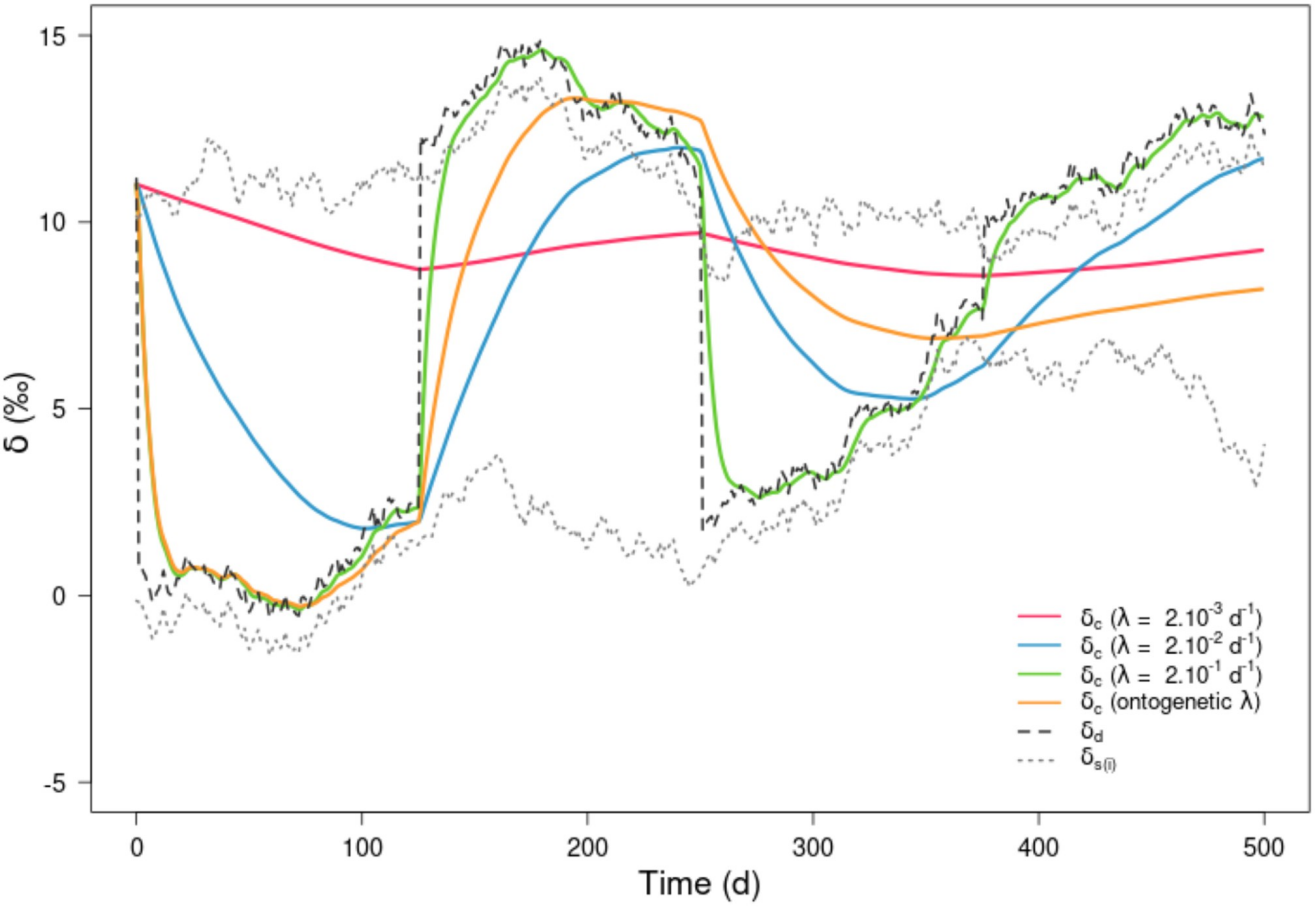
Simulated isotopic values of consumer (*δ*_*c*_(*t*)) over *T* = 500 d, for contrasted isotopic turnover rates (constant λ values in red, blue and green lines and ontogenetic λ in orange line) and experiencing a variable diet (*δ*_*d*_(*t*) in dashed dark line) resulting from four diet-switches (*ω* = 0.008 d^-1^) between two food sources ({*δ*_*s*(*a*)_; *δ*_*s*(*b*)_} in thin dashed grey lines) as simulated with Brownian trajectories. The ratios *ω*/λ were respectively 4, 0.4 and 0.04 for constant λ values and ranging from 6 to 0.04 for ontogenetic λ. Note that for each of the food source, the trophic discrimination factors (Δ_*s*(*i*)_) were set to 1 ‰.

In summary, both the deviation between *δ*_*c*_(*t*) and *δ*_*d*_(*t*) time trajectories and the time lag to reach isotopic equilibrium increased as λ decreased. If the time lag for a given λ exceeds the time when the switching takes place (in our case every 125 d) then the isotopic equilibrium could never be reached. When combined in a single metric, the simulations showed that the higher the *ω*/λ ratio the stronger the isotopic imbalance between *δ*_*c*_(*t*) and *δ*_*d*_(*t*). The inclusion of variability in the values of *δ*_*s*(*a*)_ and *δ*_*s*(*b*)_ (using Brownian trajectories) and an ontogenetic trajectory for λ provided the most complex scenario, which accumulated all components of temporal variability (Fig 3). When λ values were constant, results were quite similar to the deterministic case with four diet switches (Fig 2b). However, only the highest constant λ value (green line Fig 3) captured the Brownian variability in *δ*_*c*_(*t*). The ontogenetic λ simulation (from 2.10^−1^ d^-1^ at the beginning of the simulation to 1.10^−3^ d^-1^ at the end) brought new insights (Fig 3). At the juvenile stage in the first 80 d, λ was relatively high, so dietary switches and sources variability (*δ*_*s*(*a*)_ and *δ*_*s*(*b*)_) were rapidly integrated in the isotopic composition of consumer (*δ*_*c*_), leading to isotopic equilibrium achievement. As λ decreased, both of these features dampened, the time lag increased sharply and *δ*_*c*_ no longer reflected either the diet switch or *δ*_*s*_ values. The isotopic equilibrium could therefore not be reached. At the end of the simulation, changes in the isotopic values of the consumer are so small that variations in the sources are no longer noticeable due to a very slow turnover rate. These patterns remain valid for different Brownian trajectories of food sources isotopic values (S1 Appendix).

### Inferences in *p*-space

Contributions of the two food sources (*δ*_*s*(*a*)_; *δ*_*s*(*b*)_) to the diet (${\hat{p}}_{s(a)}$; ${\hat{p}}_{s(b)}$) of a consumer (*δ*_*c*_(*t*)) were inferred following the two methods of static mixing model (SMM_t_ and SMM_Δt_) using the previous simulations of *δ*_*c*_(*t*) derived from the dynamic mixing model (DMM). Here, only ${\hat{p}}_{s(a)}$ estimates are presented, since the sum of proportions equated to 1. The *p*_*s*(*a*)_ proportions used as reference were the ones used to force the diet in the DMM simulations. However, it should be reminded that forced *p*_*s*(*a*)_ were averaged over a time window (Δ*t*) for SMM_Δt_. Different Δ*t* proportional to the half-life (noted t_1/2_) were tested to account for the incorporation of *δ*_*d*_(*t*) into *δ*_*c*_(*t*) and a Δ*t* of twice the half-life (Δ*t* = 2 *t*_1/2_ and *t*_1/2_ = ln(2)/λ) was evidenced as the best compromise (see S2 Appendix).

**Fig 4:**
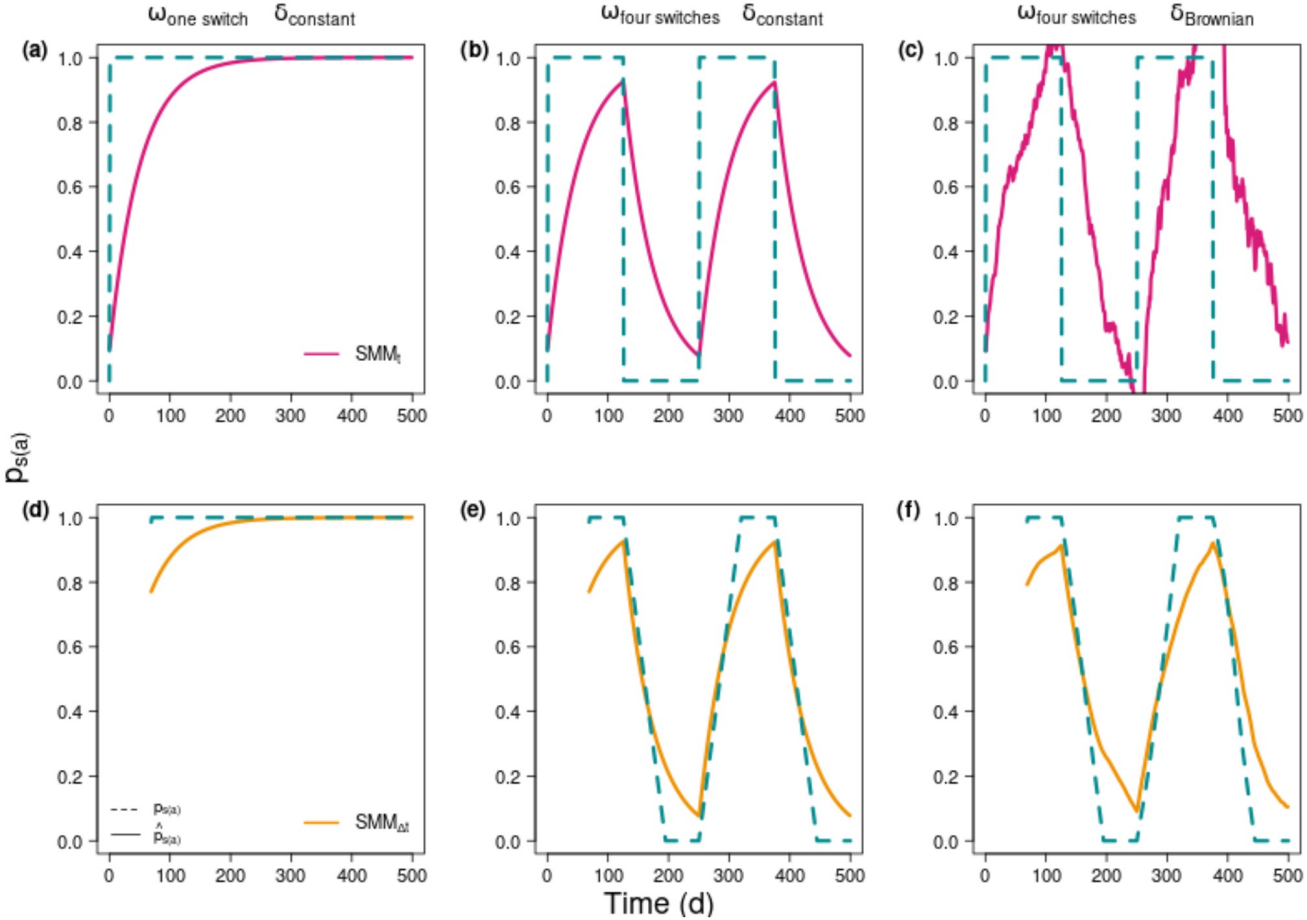
Estimated contributions of source *a* (${\hat{p}}_{s(a)}$ solid line) to a consumer’s diet compared to reference (*p*_*s*(*a*)_ dashed line). ${\hat{p}}_{s(a)}$ are inferred from isotopic composition of consumer (*δ*_*c*_(*t*)) simulated using DMM and forcing food sources (*δ*_*s*(*a*)_(*t*); *δ*_*s*(*b*)_(*t*) and *p*_*s*(*a*)_) over time. First row (a-c) represents ${\hat{p}}_{s(a)}$ estimated instantly from the SMM_t_ (pink lines), second row (d-f) represents integrative estimation of ${\hat{p}}_{s(a)}$ from SMM_Δt_ (orange lines). The reference diet (*p*_*s*(*a*)_) (turquoise dashed lines) corresponds to the forcing diet as input of DMM, at each t for SMM_t_ (a-c) or averaged over the time window (Δ*t*)—equated to twice the isotopic half-life (i.e., Δ*t* = 2 ln(2)/λ and equals 69 d for these simulations) for SMM_Δt_ (d-f). For SMM_Δt_ the ${\hat{p}}_{s(a)}$ values start at the 70^th^ day by integrating the sources over previous 69 d. The columns represent different scenarios of the experimental design: *ω* was (a, d) 0.002 d^-1^ (one diet switch), (b, c, e, f) 0.008 d^-1^ (four diet switches), and the isotopic values of the food sources were (a, b, d, e) constant or, (c, f) variable. In the three scenarios λ is constant and set at an intermediate value (λ = 2.10^−2^ d^-1^).

*p*_*s*(*a*)_ differed from ${\hat{p}}_{s(a)}$ in SMM_t_ approaches (Fig 4a–4c), and this difference drastically increased as *ω* increased and *δ*_*s*(*a*)_ varied (Fig 4a–4c). For one diet switch and constant source isotopic values (*δ*_*s*(*a*)_; *δ*_*s*(*b*)_) (Fig 4a), ${\hat{p}}_{s(a)}$ of SMM_t_ were equal to the *p*_*s*(*a*)_ after 250 d of simulation when the isotopic equilibrium was reached in *δ*-space (*δ*_*c*_(*t*) is given in Fig 2a). When *ω* increased the four inferred diet switches ${\hat{p}}_{s(a)}$ were never equal to *p*_*s*(*a*)_ (Fig 4b) and further ${\hat{p}}_{s(a)}$ was sometimes out of the diet proportion space (i.e., smaller than 0 or higher than 1) in the Brownian scenario (Fig 4c). This corresponds to situation where the consumer isotopic signature is lower than both source signatures corrected by their Δ_*s*(*i*)_ (e.g., between *t* = 350 d and *t* = 390 d on Fig 3) due to incorporation time of *δ*_*s*(*a*)_. As for the SMM_Δt_, the differences between ${\hat{p}}_{s(a)}$ and *p*_*s*(*a*)_ were significantly reduced compared to SMM_t_ (Fig 4d–4f). In addition, the improvement in the Brownian scenario circumvents the issue of being outside the *p*-space (Fig 4f). Taking into account λ via the SMM_Δt_ attenuated the effect of time lag resulting from the isotopic equilibrium assumption being at fault and more reliable dietary estimates of ${\hat{p}}_{s(a)}$ values were then obtained.

**Fig 5:**
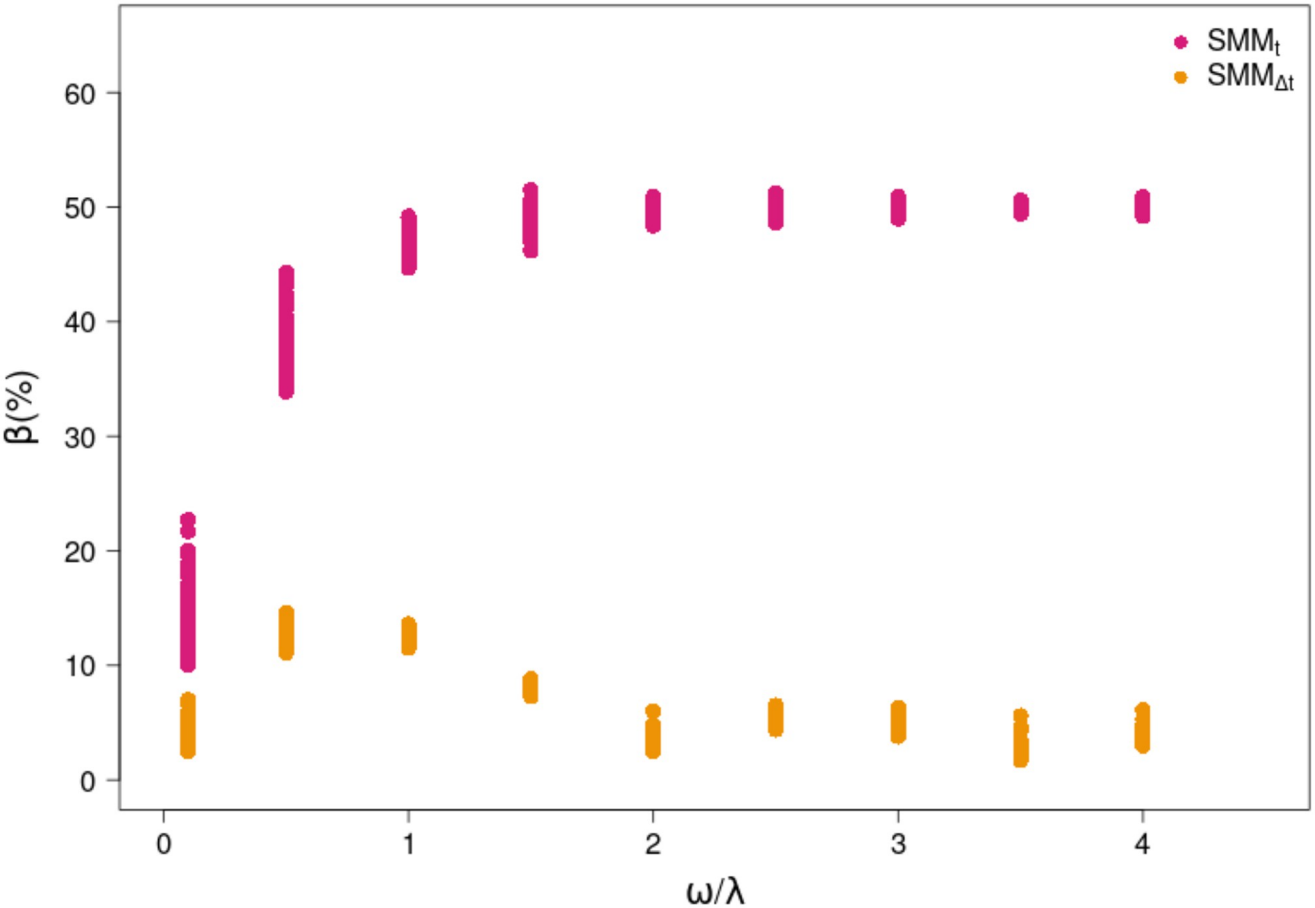
Bias estimates for the two static approaches (SMM_t_ and SMM_Δt_, pink and orange points respectively). The estimated bias ($\beta_{\frac{\omega }{\lambda }}$) for each ratio *ω*/λ is the result of the difference between forcing *p*_*s*(*a*)_ and inferred ${\hat{p}}_{s(a)}$. *ω*/λ ratio values come from combination of *ω* (2.10^−4^, 1.10^−2^, 2.10^−2^, 3.10^−2^, 4.10^−2^, 5.10^−2^, 6.10^−2^, 7.10^−2^, 8.10^−2^ d^-1^) with constant and intermediate λ value (2.10^−2^ d^-1^) to obtain a sequence of *ω*/λ between 0 and 4.

The bias ($\beta_{\frac{\omega }{\lambda }}$) estimates between SMMs and the DMM were provided for a wide range of *ω*/λ ratios (Fig 5). Although a given *ω*/λ ratio can originate from different combinations of *ω* and λ, such combinations were tested and provided similar bias value (S3 Appendix). The shapes of the bias patterns were very different between the two SMM methods (SMM_t_ and SMM_Δt_). The bias for the SMM_t_ (Fig 5, pink curve) sharply increased as the *ω*/λ ratio increased, and rapidly reached a plateau for *ω*/λ = 1. Exploration of $\beta_{\frac{\omega }{\lambda }}$ across independent values of *ω* and λ confirmed that the bias increased either when *ω* increased or when λ decreased (S3 Appendix). For SMM_Δt_ (Fig 5, orange points), the bias increased with the *ω*/λ ratio until a maximum at *ω*/λ = 0.5 and then decreased towards a slightly positive asymptote (such as an uni-modal curve with mode 0.5 and maximum bias close to 15%). The Brownian trajectories lead to a small deviation in the bias (Fig 5), which increased in low λ conditions for the same *ω*/λ ratio (S3 Appendix). Although the SMM_Δt_ was not totally dynamic and the bias was not null, the simple integration of λ greatly improved the ${\hat{p}}_{s(a)}$ estimates leading to a decrease in the bias ($\beta_{\frac{\omega }{\lambda }}$) calculation. Finally, note that the variance of values around the mean decreased when *ω*/λ increased for the two SMM methods.

### Case study

**Fig 6:**
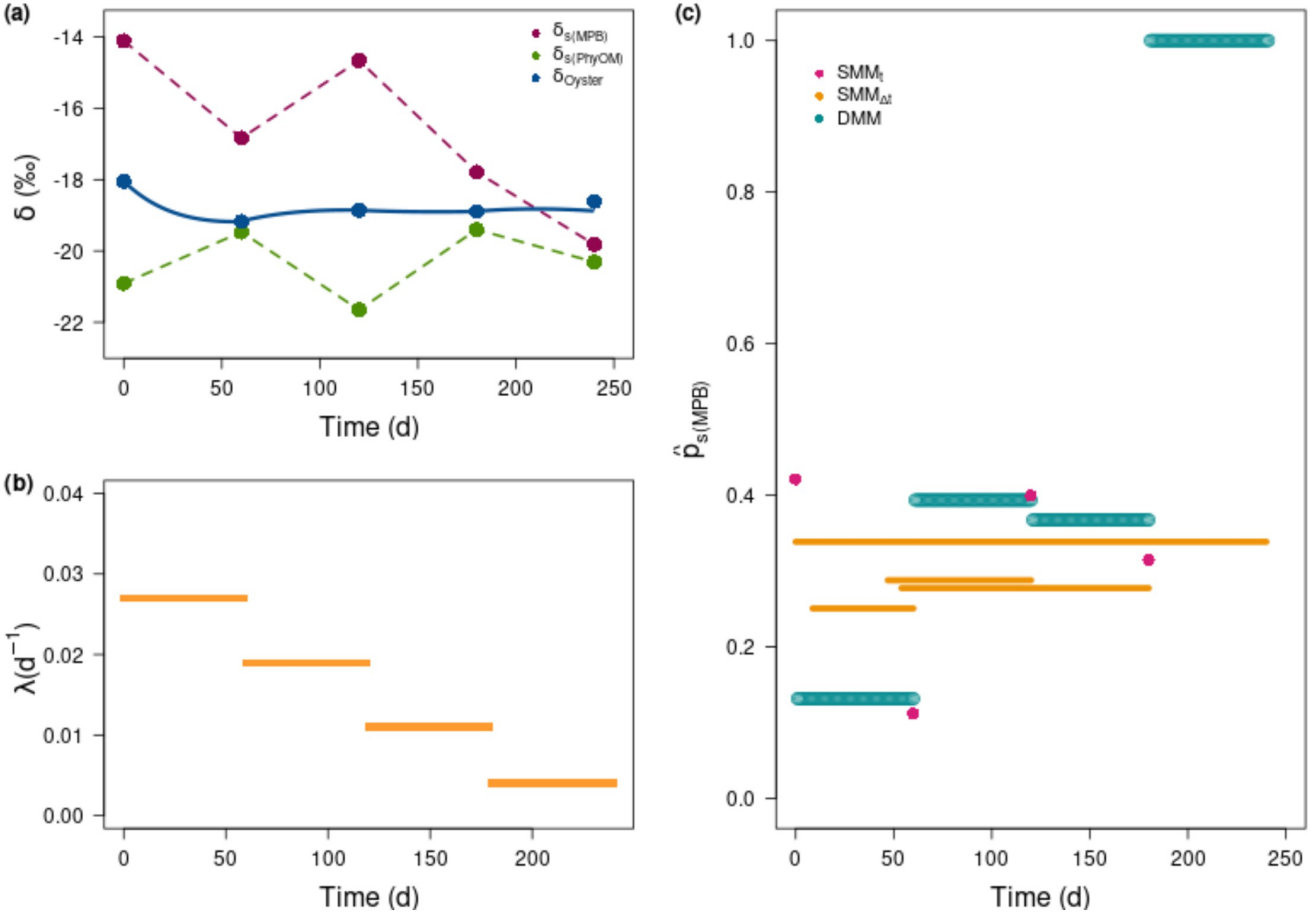
Real dataset and case study application. Data from Marín-Leal et al., (2008) [29], considering only one stable isotope (carbon). *δ*^13^C values of the consumer (Pacific oyster) and the two main sources (phytoplankton, PhyOM and microphytobenthos, MPB) once corrected by their respective Δ_*s*(*i*)_ (here equal to 1 ‰) and their linear interpolation between sampling dates (a). Estimated λ values for each sampling dates (b). Estimates of microphytobenthos contributions (${\hat{p}}_{s(MPB)}$) to the oyster’s diet, according to different methods (c). Instantaneous SMM_t_ is represented by pink dots at sampling date, integrated SMM_Δt_ used averaged sources over a time window of two half-lives, and therefore estimates ${\hat{p}}_{s(MPB)}$ constant over this time window. Note that with decreasing λ, the time window increases, resulting in longer orange bars (constant ${\hat{p}}_{s(MPB)}$ estimated over a longer period). Furthermore, since the last window is a bit larger than the sampling period by 106 d, the entire period of sampling was considered. ${\hat{p}}_{s(MPB)}$ is estimated through DMM for each period between sampling dates (turquoise bars).

Data from Marín-Leal et al. [29] were used as a case study to contrast the estimates of source proportion to the diet obtained with the three methods: SMM_t_, SMM_Δt_ and DMM. In this dataset, there were two food sources, the *δ*^13^C values of which varied over time: particulate organic matter (PhyOM) and microphytobenthos (MPB). The *δ*^13^C values of PhyOM fluctuated between -19 ‰ and -22 ‰ (once corrected by Δ_*s*(*PhyOM*)_ = 1 ‰). The *δ*^13^C values of MPB decreased over time from -14 ‰ to -20 ‰ (once corrected by Δ_*s*(*MPB*)_ = 1 ‰), with fluctuations (Fig 6a). Isotopic values of both food sources (*δ*_*s*_) were distinct except for the last sampling date where the Δ_*s*_-corrected value of MPB decreased and approached the Δ_*s*_-corrected value of PhyOM (at approximately -20 ‰, i.e., at t = 240 d *δ*_*s*(*MPB*)_ = −19.82 ‰ and *δ*_*s*(*PhyOM*)_ = −20.31 ‰). The consumer (i.e., oyster) *δ*^13^C values displayed low variability through time, with an initial 1 ‰ decrease then followed by steady values around -19 ‰. Oyster λ estimates decreased over time, from 0.027 d^-1^ to 0.004 d^-1^. It is worth noticing that the *δ*^13^C value of the consumer was outside the isotopic polygon of the two food sources at the last sampling date. Then, SMM_t_ could not be applied at this last point (this is a requirement of stable isotope mixing models) and only SMM_Δt_ and DMM could provide ${\hat{p}}_{s(MPB)}$ estimates.

The three mixing models produced different diet estimates (Fig 6c). Although the SMM_Δt_ and DMM methods gave similar increasing trends, the ranges were different (less pronounced for the integrated SMM_Δt_). Estimated ${\hat{p}}_{s(MPB)}$ varies from 0.25 to 0.34 for SMM_Δt_ and from 0.13 to 1 for DMM over the four periods. For SMM_t_, estimations of ${\hat{p}}_{s(MPB)}$ fluctuated between 0.42 and 0.11 without a clear pattern. In this case, ${\hat{p}}_{s(MPB)}$ were only the result of the *δ*^13^C values of the consumer within the polygon of the *δ*^13^C values of the food sources. For SMM_Δt_, the integration time window (i.e., equal to twice the half-life) had a buffering effect on the estimates and dampened the variability of the ${\hat{p}}_{s(MPB)}$ estimates between the different periods, which results from λ decreasing over time. DMM was able to reproduce the trajectories of the oyster *δ*_*c*_(*t*) adequately by tuning ${\hat{p}}_{s(MPB)}$; i.e., simulated *δ*_*c*_(*t*) were similar to sampled *δ*_*c*_(*t*) except for the last sampling point where the best DMM estimate of *δ*_*c*_(*t*) was at 0.27 ‰ below the observed one. Between the first two sampling dates, the consumer’s isotopic value (*δ*_*c*_(*t*)) changed significantly, which explains the small discrepancy between SMM_Δt_ and DMM. While both SMM_Δt_ and DMM captured the temporal variability of food sources isotopic values, only DMM accounted for those of the consumer. The gap among estimates was large for the fourth period since DMM accounted both for the *δ*_*s*(*i*)_(*t*) trajectories and the ones of *δ*_*c*_(*t*), but with a small λ (0.004 d^-1^, Fig 6b), while SMM_Δt_ averaged the *δ*_*s*(*i*)_(*t*) over the eight months, and SMM_t_ could not provide a solution because the consumer was outside the food source polygon in the real *δ*-space (Fig 6a). DMM is the only method that takes into account all the dynamics (λ(t), *δ*_*s*(*i*)_(*t*) and *δ*_*c*_(*t*)) and can therefore provide a reasonable estimate (Fig 6c) even if the consumer i) had no distinct values with its isotopic sources for a short period or ii) was outside the isotopic polygon of food sources (considering only two sources, with a sufficient sampling period for a low λ involving a long time lag).

## Discussion

Animal diets often change with time: vagile animals move across isoscapes (e.g., Trueman et al. [53]) and sessile organisms face change in food availability depending on seasons (e.g., Kaufman et al. [54]; Marín-Leal et al. [29]). For isotopic ecologists and physiologists eager to understand animal diets, the purpose of a dynamic mixing model (DMM) is to account for all temporal variabilities within a consumer’s incorporation dynamics: isotopic values of food sources *δ*_*s*_(*t*) and of the consumer *δ*_*c*_(*t*), frequency of diet-switch (*ω*), expressed in source proportions to the diet *p*_*s*_(*t*), and isotopic turnover rate λ(*t*). The DMM approach is based on isotopic processes set in a mechanistic framework. The quantification of bias produced by conventional inferential methods (i.e., the static mixing model, SMM) compared to the simulations provided by the DMM approach using an *in-silico* experiment highlighted the weaknesses of SMM methods that rely on the strong assumption of an isotopic equilibrium between a consumer and its prey. A systematic bias—always presented as consequence of strong assumptions—was observed in instantaneous SMM (SMM_t_), considering only isotopic values of food sources (*δ*_*s*_) and consumer (*δ*_*c*_) as a snapshot. By considering a time window (Δ*t*) inversely proportional to λ of consumer, applying integrated SMM (SMM_Δt_) is a first step to reduce such a bias. However, SMM_Δt_ is limited and enables to estimate an average diet over a time period (i.e., a period of several snapshots). Only DMM takes into account the time lag needed for a prey to be integrated in consumer’s tissues. Accounting for the isotopic turnover rate (λ) of consumer is therefore crucial for i) capturing dietary variations, ii) improving diet estimates (*p*_*s*_) and iii) relaxing the strong isotopic equilibrium assumption.

### Improvement of diet estimates implies the consideration of several dynamics

In trophic ecology, diet reconstruction and trophic level estimates are probably the most common uses of stable isotope-based tools to investigate species’ diet and food-web structure [20, 55]. Diet estimates are often deduced from a single snapshot of isotopic values sampled over space or time [56]. Since inferences in isotopic ecology rely on the isotopic equilibrium assumption, they can induce a misinterpretation of ecological processes [30, 57]. However, determining adequate time windows for sampling food sources and isotopic turnover rate (λ) of tissues is a well-known issue in isotopic ecology, highlighting that time is a critical component for all stable isotope mixing model studies [9, 31]. To date, the method used to track diet changes over time is the isotopic clock, which consists of analysing several tissues with different λ values, allowing back-calculation of diet at different time windows and so improving the accuracy of diet reconstructions [20]. For example, using an isotopic clock, MacNeil et al. [58], Heady & Moore [59] and Shipley et al. [34] showed seasonal dietary shifting for blue shark (*Prionace glauca*), for rainbow trout (*Oncorhynchus mykiss*) and sand tiger shark (*Carcharias taurus*) respectively. However, only the diet switches circumscribed in time can be detected with this method under the assumption of constant λ and trophic discrimination factor (Δ_*s*(*i*)_) over the time window of sampling.

**Fig 7:**
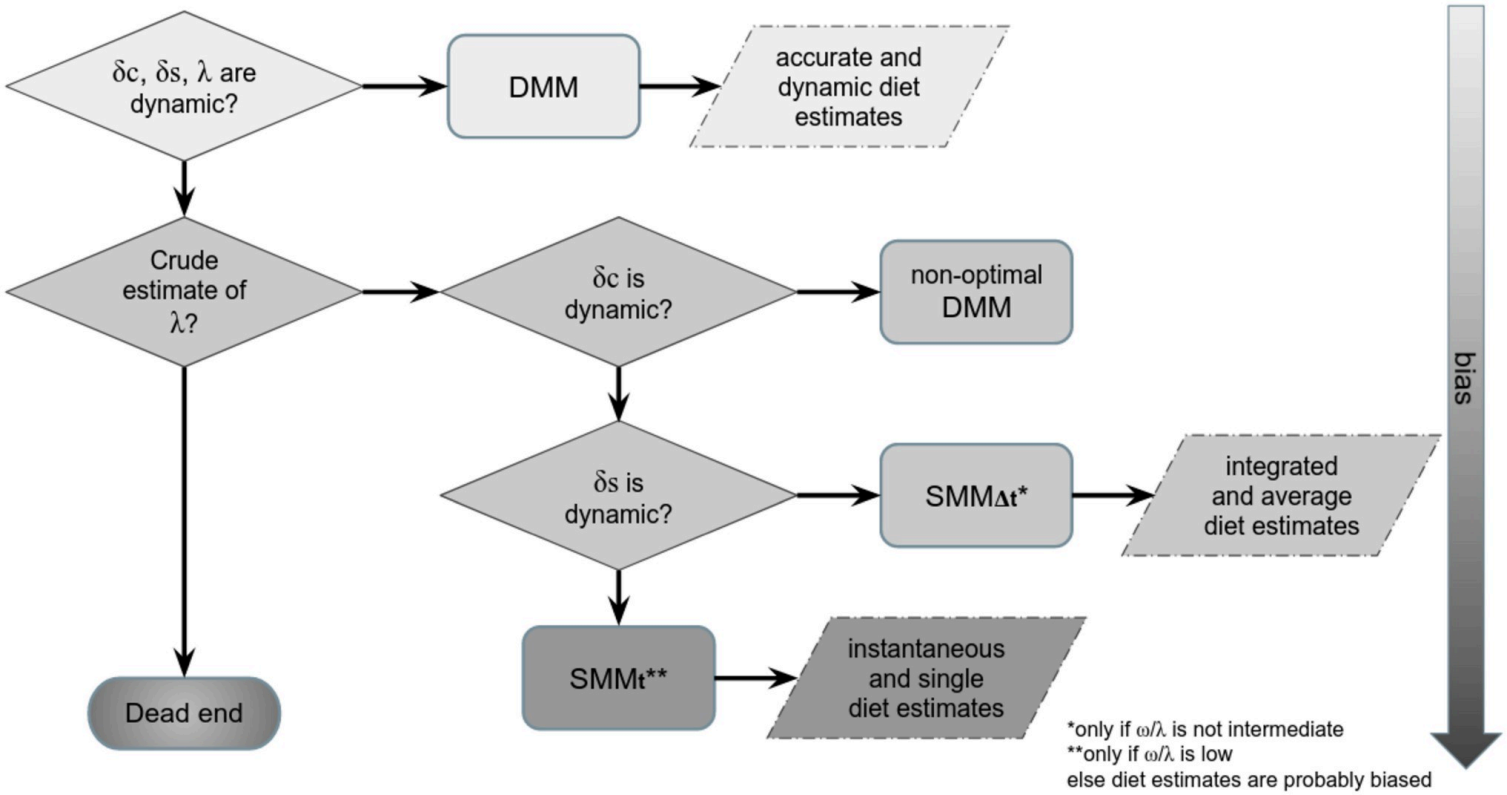
Decision tree of the most suitable method for estimating source proportions to the diet of one consumer considering the dynamics of λ and *δ* values, to reduce the bias induced by the isotopic equilibrium assumption. The diagram highlights the key aspects to be considered on the estimation of λ, the sampling of consumer and food source dynamics in order to determine which model can be applied (SMMs or DMMs).

Although isotopes integrate natural fluctuations in space and time [1], a dynamic estimation of the diet is necessary to better picture the temporal scale of diet variations for one consumer [57]. In that perspective of temporal insight, inferences drawn from DMM outperform those derived from SMM_Δt_ by integrating all dynamics together (*δ*_*c*_(*t*), *δ*_*s*_(*t*), *ω* and λ(*t*)) and by providing a dynamic diet estimate as evidenced in our case study results. The improvement of diet estimates depends on DMM’s capacity to reduce the bias resulting from erroneously assuming the isotopic equilibrium assumption in SMM methods. Results highlight that λ and dietary shifting (*ω*) have a strong impact on diet contribution estimates (Fig 7). Hence, our results showed that the instantaneous approach (i.e., SMM_t_) failed to estimate diet as soon as *ω* increased or λ decreased. For a ratio *ω*/λ greater than 1, the bias ($\beta_{\frac{\omega }{\lambda }}$) in diet estimates was in the order of 50% (for the wide range of explored *ω* and λ values). However, SMM_t_ may provide reasonable diet estimates when *ω*/λ is close to zero (Fig 7) but at the expense of a higher variance—which depends on the combination between the variance of Brownian sources and the relatively low λ values to obtain a ratio close to zero. In this situation, the isotopic values of the consumer converge towards the isotopic equilibrium with prey. Assuming isotopic equilibrium may only be reasonable when using tissues associated to a high λ (e.g., plasma), and low *ω* (e.g., an exclusively specialist organism) and only if isotopic values of the food sources are quite constant (Fig 7). In other words, focusing on high turnover tissues of a specialist predator with prey having a constant isotopic value is almost impossible and points out the crucial importance of considering λ. Following the recommendation of Phillips et al. [9], the SMM_Δt_ has the advantage of reducing $\beta_{\frac{\omega }{\lambda }}$ to a maximum value of 15% by considering a time window—inversely proportional to λ—over which isotopic values of the food sources (*δ*_*s*_) are averaged. However, a strong variability in the *δ*_*s*_ increased the variance of the bias in diet estimates when *ω*/λ decreased, a result also found by Yeakel et al. [41] for the variance of the isotopic niche with *ω*/λ ratios from 0.5 to 2.

The bias generated by SMM_Δt_ is then a unimodal function of the *ω*/λ ratio (peaking at values around 0.5), which is revealed in the natural environment by the frequency of diet-switch between preys (e.g., availability of prey, switching of food sources in a new habitat after migration), and/or changes in the metabolism of the organism over its life cycle (e.g., ontogenetic shifting, growth rate).

A dietary-specialist species always feeds on the same diet (composed of several prey but remains fixed over time), while a generalist species feeds on a varying diet (changing of consumed prey over time) switching quickly from one to another [56, 60]. The biases in their respective dietary estimates are placed at the extreme ranges of the *ω*/λ values (Fig 5). However, the situation differs depending on which trophic level (TL) specialist or generalist consumers are foraging. Isotopic values of primary producers vary strongly depending on environmental gradients [23, 24] and their isotopic temporal variabilities propagate upwards in the food web but dampen in higher trophic levels [40]. Generalist species at intermediate or high TLs (*TL* > 3) displaying slowly changing isotopic values, and switching their diet very frequently on numerous prey are good candidates for SMM_Δt_. This is for example, the case for large omnivorous adult fish species [61] or crabs foraging on a large diversity of macro-invertebrates [62], as long as all isotopic values of potential prey are taken into account over the appropriate time window. Nevertheless, the DMM would be necessary to estimate accurately the moment of habitat change if migration occurs [34, 62, 63]. For specialist consumers foraging at TL lower or equal to 3, the issue for determining diet stands in the temporal variability of isotopic values of food sources, which cannot be fully captured without a DMM approach and an accurate estimation of λ. This is for example, the case of blue whales migrating over large spatial scales [53] or migratory specialist seabirds with large foraging grounds [64]. However, for specialist top predators such as polar bears, which feed mainly on higher trophic level prey (e.g., seals [65]) characterized by low temporal variability of their *δ*_*s*_ (annually ∼ 1 ‰ on both *δ*^15^N and *δ*^13^C values [66]) the use of a SMM_t_ might still yield appropriate dietary estimates if tissues with high λ are sampled (e.g., plasma).

The peak of $\beta_{\frac{\omega }{\lambda }}$ corresponds to intermediate dietary shifting and turnover rate when the isotopic values of the consumer (*δ*_*c*_) were in transition (i.e., far from isotopic equilibrium) after a diet switch, so the isotopic trajectory of *δ*_*d*_(*t*) is continuously changing and the isotopic equilibrium is never reached [41]. The use of DMM is then highly relevant (Fig 7) and avoids questions about the conditions of SMMs application. For example, isotopic turnover of muscle tissue in ectotherms ranged from 0.2 to 0.002 d^-1^ [31]. This means, more specifically, that the bias peaked for frequency of diet-switch between 10 and 1000 days. This time window typically corresponds to seasonal or spatial changes in subsidies and/or ontogenetic diet changes in moderate and large size animals with lifespan ranging from several years to several decades, respectively. This is the case of generalist consumers changing prey at moderate frequencies in marine and terrestrial ecosystems, because of the variability of the prey abundance [29, 67–69] or ontogenetic shift in the diet [70].

### How to estimate λ?

Despite the exponential use of stable isotope ratios in trophic ecology over the last two decades, the “fruitful, and perhaps urgent, task” [22] to estimate and integrate the temporal dynamic of isotopic incorporation is still vastly overlooked. Yet, an accurate estimation of the isotopic turnover rate (λ) is required to operate the DMM and, to some extent, more conventional approaches such as SMM_Δt_. To date, λ has been estimated using experimental and/or modelling methods. Experimentally, λ is estimated by labelling and tracking a chemical element over time, such as *δ*^13^C or *δ*^15^N [20]. In diet-switch experiment, λ is estimated by fitting a model describing the isotopic incorporation dynamics of the target animal to isotopic observations, typically the time model [46] or the mass model [36]. λ is one explicit parameter in the exponential component of the time model and represents the necessary time for an individual to achieve the isotopic equilibrium with its new consumed diet in experimental conditions [3]. The diet-switch experiment method requires measurements over time of isotopic values of a consumer raised in laboratory conditions. For example, Guelinckx et al. [71] conducted an experiment on sand goby (*Pomatoschistus minutus*) and analysed three tissues: heart, liver and muscle for both carbon and nitrogen stable isotopes, to build an isotopic clock using λ. Nevertheless, performance of diet-switch experiment requires to study animals which i) are able to adapt to captivity conditions well, ii) grow and reach an isotopic equilibration with their experimental diet reasonably quickly and, iii) are not endangered or under any legal restrictions preventing any manipulative experiments. An alternative method to estimate λ is to transform the isotopic half-life (*t*_1/2_) registered thanks to diet-switch experiment for many animals species and tissue types of animals in the meta-analyses done by Thomas & Crowther [31], and by Vander Zanden et al. [17]. These meta-analyses quantify the link between λ and body mass [17], and also temperature [31]. Indeed, λ scaled with body mass power 0.2; a value close to the originally predicted 0.25 at the species level [51]. Actually, λ is assumed to be the sum of two rates: growth and catabolic rates [32]. These two rates vary with time as a function of environmental drivers and physiological status, and this involves a dynamic λ of an organism during its lifespan. For example, Herzka [37] assumed that growth was dominant mechanism over catabolism driving λ in young fishes. Consequently, she neglected the catabolic rate and focused on the estimation of the growth rate only to predict λ. In a more developed approach, Guelinckx et al. [38] combined field-measured instantaneous growth rate and metabolic rate as measured by mass specific oxygen consumption to estimate a variable λ. Finally, some studies made use of bioenergetic modelling approaches to efficiently account for dominant physiological processes involved in λ. In one of the first application, Harvey et al. [40] referred to a bioenergetic model to estimate the growth rate and body mass dynamics and then λ over time, by adding a fixed catabolic rate [40]. The same approach was used later by Weidel et al. [72]. In parallel, dynamic energy budget models (a mechanistic version of bioenergetic models) were considered to assess the value of λ more directly, by accounting simultaneously for the growth and catabolic rates [29]. In this vein, dynamic isotopic models were then developed [16, 73] and represent the most sophisticated models of isotope incorporation so far. In the latter, λ and the trophic discrimination factor are no longer parameters of the model but outputs combining many physiological processes.

Isotope dynamics result from the combination of the dilution effect through incorporation rate (λ) and discrimination events (i.e., causing the trophic discrimination factor (noted Δ_*s*(*i*)_)) [39]. Besides λ, the trophic discrimination factor is recognised as a critical parameter in stable isotope mixing models. Δ_*s*(*i*)_ estimates and the various factors that impact on its variations (isotope element, tissue, diet, excretion form, habitat, metabolism, amino-acid in protein, phylogeny, sex) have been reviewed in multiple studies [11–13, 74–78]. In mixing models, the trophic discrimination factor is assumed independent from λ and often only a mean value is used for one isotope element and for all studied organisms. In our approach, Δ_*s*(*i*)_ was considered constant and equal to 1 ‰ for both sources to highlight the impact of λ. Changing values for Δ_*s*(*i*)_ would impact the absolute estimation of the diet at one given time but not the relative estimation from one time to another one. This has been shown by several papers displaying sensitivity analyses on Δ_*s*(*i*)_ values [29, 79]. In the framework of DMM, one step forward would be to consider the time dynamics of Δ_*s*(*i*)_. The isotopic incorporation rate—which depends on λ—may co-vary negatively with the Δ_*s*(*i*)_ as shown experimentally by [80], Gorokhova [81] or Lefebvre & Dubois [82], and theoretically by Emmery et al. [16]. Alternatively, a solution to estimate dynamically trophic discrimination factor and λ in the meantime is to use the IsoDyn model, a kinetic one-compartment model based on isotopic fluxes and body mass dynamics in an organism considering the isotopic processes in body mass gains and losses [83]. Formalizing the inter-connection between these two key parameters (λ and Δ) could be a key perspective to improve the stable isotope mixing models.

### Further uses of DMM and perspectives beyond diet reconstruction

The bias resulting from neglecting λ dynamics that we estimated may be considered in stable isotope mixing model methods. The Bayesian framework integrates fully and explicitly the uncertainty associated with multiple food sources, discrimination factors and isotope signatures [45], but not in a dynamic way. The inclusion of informative priors from conventional methods in Bayesian mixing models can transfer biases into model outcomes, leading to erroneous results [7]. However, Bayesian stable isotope mixing models are able to infer the relative importance of food sources in wild animal diets, when accurate estimates of parameters and priors are used. Improving diet estimates in a Bayesian framework can be done in two ways. First, the use of λ would allow to determine the time window over which uncertainty of food sources has to be evaluated and sampled. Lastly, posteriori distributions provide a “modal indication” of the estimate of the consumer’s diet. In this case it is still a correct average estimate for a defined sampling window but does not reflect trophic changes in the system. Implementing explicit dynamics via DMMs into a Bayesian framework would be an interesting perspective.

Another use of DMMs stands in the determination of diet specialisation in individuals and ultimately in the trophic niche width estimates of the population as measured by the isotopic variance between individuals [56, 84]. In an experimental approach, Fink et al. [60] showed that the isotopic variance depended on the dietary correlation time, i.e., the time window over which the consumer changes its diet. Later, Yeakel et al. [41] evidenced the role of λ and prey switching frequency (*ω*) in the peak of variance when consumers are in transition between two diets. λ scales allometrically with body mass [31] and is proportional to food incorporation rate [51]. λ should then vary within and between individuals and its variation should participate to the isotopic variance independently of diet as suggested by Hette-Tronquart [85]. Compared to Yeakel’s analytical equation [41], the numerical solution of the DMM developed here offers the advantage to vary λ, and to back-calculate dynamic diets. Improving estimation of individual diet specialisation using the DMM would require hard tissues (e.g., baleen in whales [53], scales or otoliths in fish [86], shells in bivalves [87]) to be analysed both to record isotopic values of the consumer and to estimate growth increment and metabolism over time. Finally, Flynn et al. [39] and Trueman et al. [53] advocated the use of simulation modelling to challenge respectively the trophic level via *δ*^15^N and to reconstruct migration patterns in whales. The isotopic niche must consider dynamics of consumer foraging behaviours, changing in response to prey availability, temporal changes in the environment as seasonality, ability of the consumer to find, acquire, and consume its prey, intraspecific competition and physiological state [41]. Then, another way to explore isotopic variance would be to incorporate the DMM within a dynamic population model which uses the individual as the fundamental unit, and where the dynamics are governed by individual rules for growth, movement, reproduction, feeding, and mortality [88] and ultimately in end-to-end models [89] to fully consider all trophic interactions within a community along environmental gradients [42].

## Conclusion

The isotopic incorporation dynamics of consumers are mainly driven by the isotopic turnover rate which leads to a time lag in the isotopic equilibrium between the isotopic values of the consumer (*δ*_*c*_) and its diet (*δ*_*d*_). The dynamic mixing model (DMM) accounts for all the dynamics of the three factors (isotopic values of both the food sources (*δ*_*s*_) and of the consumer (*δ*_*c*_) and isotopic turnover rate (λ)) used as forcing variables to determine the diet, although it has not included yet the underlying processes driving the isotopic value of the food sources or the isotopic turnover rate of the consumer (such as growth and physiological state). Using an instantaneous static mixing model (SMM_t_, Fig 7) should be restricted to specific species (e.g., specialist top predators) and to some sampled tissues (e.g., plasma). Considering a time window of integration (Δ*t*) proportional to the isotopic half-life improves the diet estimates in integrated static mixing model (SMM_Δt_), which is suitable in most cases. Nevertheless, the SMM_Δt_ method provides only an averaged diet of one consumer over Δ*t* and still requires both an accurate average estimation of the isotopic turnover rate over the time window under study and the evolution of isotope values of the food sources over this same window (Fig 7). DMM offers more accurate diet estimates over time but requires further sampling efforts regarding the evolution of the isotopic values of the consumer as well as an estimation of the isotopic turnover rate over time (Fig 7). Applications of DMM could be generalized to the study of isotopic niches, in which an unestimated and significant part of the niche variance is probably due to not taking into account isotopic variabilities. Finally, we argue that isotopic dynamics may be implemented in future studies as additional state variables in ecosystem models.

## Supporting information

S1 Appendix Patterns of simulated isotope values of consumer for different scenarios of λ values remain valid for different Brownian trajectories of food sources signatures. Trajectories of simulated consumer (*δ*_*c*_) over time for the 4 scenarios of isotopic turnover rate (λ in d^-1^): low λ (red line, a), medium λ (blue line, b), high λ (green line, c), ontogenetic λ (orange line, d) and *ω* = 0.008 d^-1^(equivalent to 4 diet-switches) from 10 first Brownian sources simulations among 100 simulations (e) for both food sources: source a (solid line) and source b (dashed line). Whatever the pair {*δ*_*s*(*a*)_; *δ*_*s*(*b*)_} used in DMM forcing, the same patterns are observed in *δ*_*c*_ for each given scenario of {*ω*; λ} in our *in-silico* experiment.
(PDF)

S2 AppendixChoice of integration window length for the integrated static mixing model.Representation of bias estimates ($\beta_{\frac{\omega }{\lambda }}$) as a function of the *ω*/λ ratio for the instantaneous (SMM_t_) and integrated (SMM_Δt_) methods. For SMM_Δt_, four lengths of integration window (Δ*t*) were tested, such as Δt equals to half a time, once, twice, three times the half-life (i.e., 0.5 × *t*_1/2_, 1 × *t*_1/2_, 2 × *t*_1/2_, 3 × *t*_1/2_). The most appropriated Δt to improve the diet estimates by integration method corresponds to the best compromise to reduce $\beta_{\frac{\omega }{\lambda }}$. The selected Δt for the manuscript is Δ*t* = 2 × *t*_1/2_.(PDF)

S3 AppendixExploring the bias estimates ($\beta_{\frac{\omega }{\lambda }}$) for different combinations of *ω* and λ.Additional frequency of diet switch (*ω*) and turnover rate (λ) values (in the Table S3.A) are used to provide different scenarios of simulated isotopic value of consumer (*δ*_*c*_). From *δ*_*c*_ different methods of static mixing model (SMM) are applied and the bias estimates ($\beta_{\frac{\omega }{\lambda }}$) allow to compare them. Before the use of the metric *ω*/λ a preliminary test is carried out to explore the respective effects of *ω* and λ on $\beta_{\frac{\omega }{\lambda }}$, using a bubble plot (see the first graph S3.B). The bubble plot is applied only on instantaneous static mixing model (SMM_t_) with constant food sources (*δ*_*s*_) over time. To explore also the impact of dynamic *δ*_*s*_ over time and the integrated static mixing model (SMM_Δt_) method a pseudo-sensitivity analysis is conducted (in the second graph S3.C).(PDF)
