# Supplementary material for: Inferences to estimate consumer’s diet using stable isotopes: Insights from a dynamic mixing model
Source: PLoS One. 2022 Feb 7;17(2):e0263454. doi: 10.1371/journal.pone.0263454 (PMC8820609; doi:10.1371/journal.pone.0263454)

**S1 Appendix.** Patterns of simulated isotope values of consumer for different scenarios of $\lambda$ values remain valid for different Brownian trajectories of food sources signatures.

Trajectories of simulated consumer ($\delta_c$) over time for the 4 scenarios of isotopic turnover rate ($\lambda$ in $d^{-1}$): low $\lambda$ (red line, a), medium $\lambda$ (blue line, b), high $\lambda$ (green line, c), ontogenetic $\lambda$ (orange line, d) and $\omega = 0.008$ $d^{-1}$(equivalent to 4 diet-switches) from 10 first Brownian sources simulations among 100 simulations (e) for both food sources: source a (solid line) and source b (dashed line). Whatever the pair $\{\delta_{s(a)}$ ; $\delta_{s(b)}\}$ used in DMM forcing, the same patterns are observed in $\delta_c$ for each given scenario of $\{\omega;\ \lambda\}$ in our *in-silico* experiment.

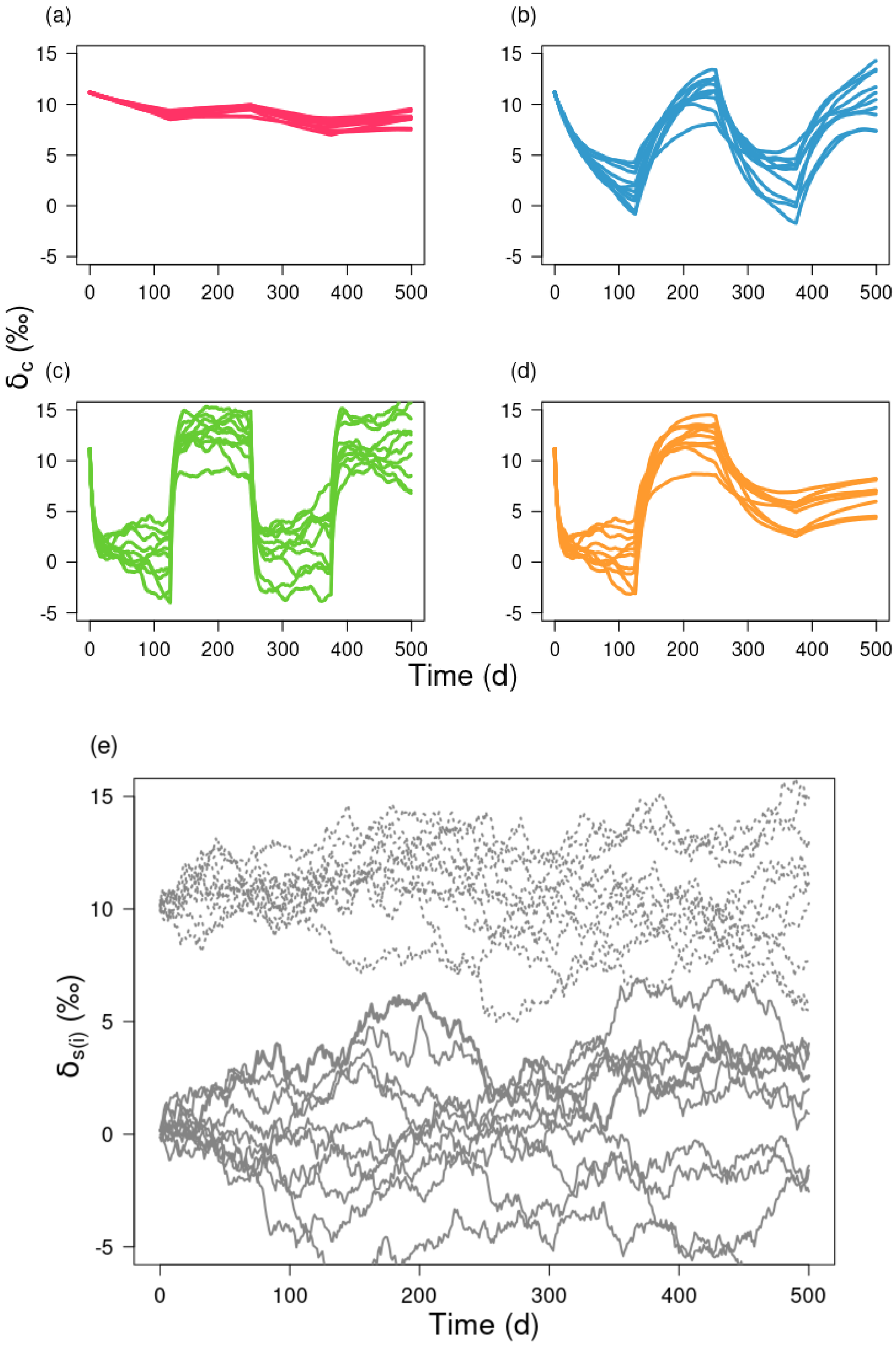

Supplement: S1 Appendix — Trajectories of simulated consumer (δc) over time for the 4 scenarios of isotopic turnover rate (λ in d-1): low λ (red line, a), medium λ (blue line, b), high λ (green line, c), ontogenetic λ (orange line, d) and ω = 0.008 d-1(equivalent to 4 diet-switches) from 10 first Brownian sources simulations among 100 simulations (e) for both food sources: source a (solid line) and source b (dashed line). Whatever the pair {δs(a); δs(b)} used in DMM forcing, the same patterns are observed in δc for each given scenario of {ω; λ} in our in-silico experiment. (PDF) [file pone.0263454.s001.pdf]
