# Supplementary material for: Inferences to estimate consumer’s diet using stable isotopes: Insights from a dynamic mixing model
Source: PLoS One. 2022 Feb 7;17(2):e0263454. doi: 10.1371/journal.pone.0263454 (PMC8820609; doi:10.1371/journal.pone.0263454)

S2 Appendix. Choice of integration window length for the integrated static mixing model.

Representation of bias estimates ($\beta_{\frac{\omega}{\lambda}}$) as a function of the ω/λ ratio for the instantaneous (SMM_t) and integrated ($SMM_{\Delta t}$) methods. For $SMM_{\Delta t}$, four lengths of integration window (Δt) were tested, such as Δt equals to half a time, once, twice, three times the half-life (i.e., $0.5 \times t_{1/2}$, $1 \times t_{1/2}$, $2 \times t_{1/2}$, $3 \times t_{1/2}$). The most appropriated Δt to improve the diet estimates by integration method corresponds to the best compromise to reduce $\beta_{\frac{\omega}{\lambda}}$. The selected Δt for the manuscript is $\Delta t = 2 \times t_{1/2}$.

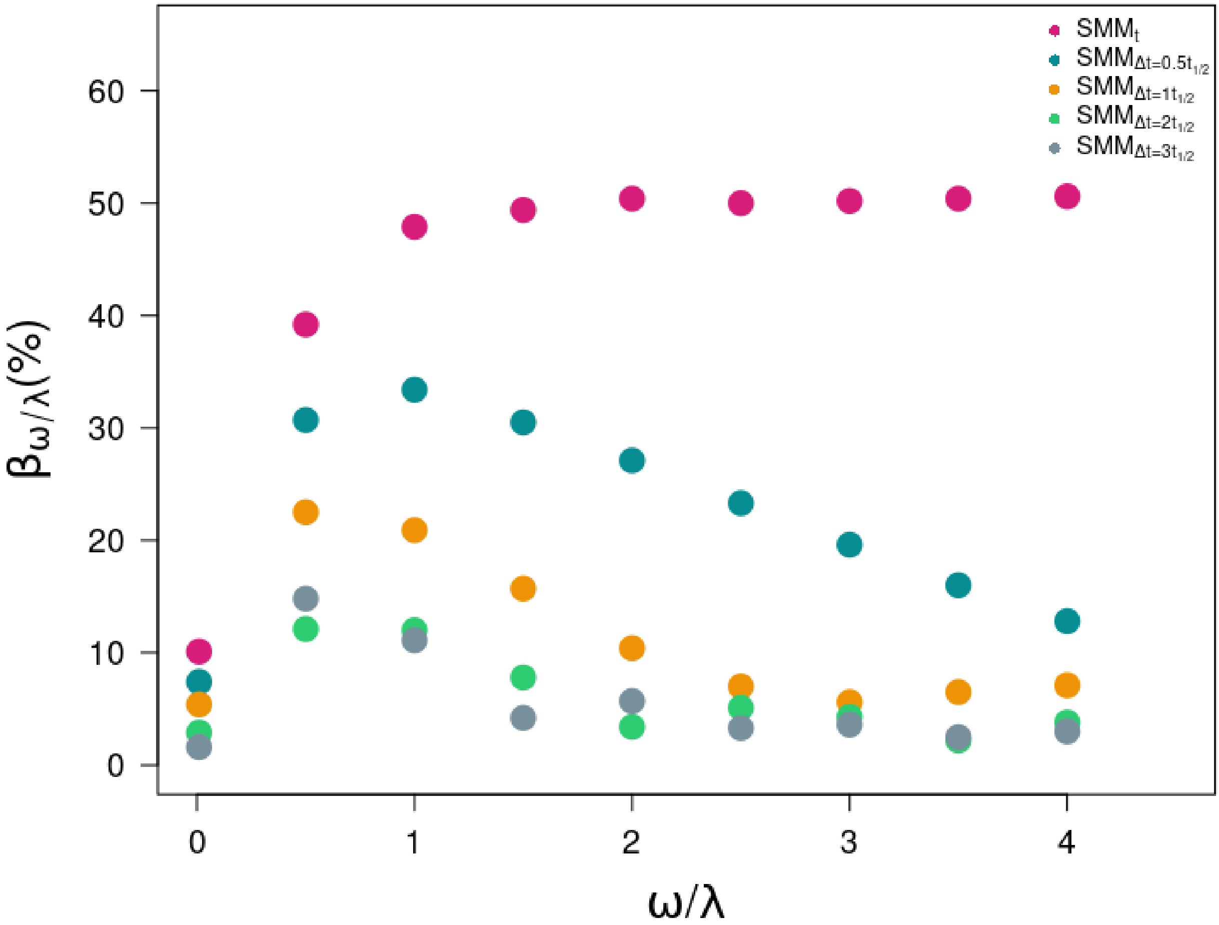

Supplement: S2 Appendix — Representation of bias estimates (βωλ) as a function of the ω/λ ratio for the instantaneous (SMMt) and integrated (SMMΔt) methods. For SMMΔt, four lengths of integration window (Δt) were tested, such as Δt equals to half a time, once, twice, three times the half-life (i.e., 0.5 × t1/2, 1 × t1/2, 2 × t1/2, 3 × t1/2). The most appropriated Δt to improve the diet estimates by integration method corresponds to the best compromise to reduce βωλ. The selected Δt for the manuscript is Δt = 2 × t1/2. (PDF) [file pone.0263454.s002.pdf]
