# Supplementary material for: Inferences to estimate consumer’s diet using stable isotopes: Insights from a dynamic mixing model
Source: PLoS One. 2022 Feb 7;17(2):e0263454. doi: 10.1371/journal.pone.0263454 (PMC8820609; doi:10.1371/journal.pone.0263454)
Supplement: S3 Appendix — Additional frequency of diet switch (ω) and turnover rate (λ) values (in the Table S3.A) are used to provide different scenarios of simulated isotopic value of consumer (δc). From δc different methods of static mixing model (SMM) are applied and the bias estimates (βωλ) allow to compare them. Before the use of the metric ω/λ a preliminary test is carried out to explore the respective effects of ω and λ on βωλ, using a bubble plot (see the first graph S3.B). The bubble plot is applied only on instantaneous static mixing model (SMMt) with constant food sources (δs) over time. To explore also the impact of dynamic δs over time and the integrated static mixing model (SMMΔt) method a pseudo-sensitivity analysis is conducted (in the second graph S3.C). (PDF) [file pone.0263454.s003.pdf]

S3 Appendix. Exploring the bias estimates ($\beta_{\frac{\omega}{\lambda}}$) for different combinations of ω and λ.

Additional frequency of diet switch (ω) and turnover rate (λ) values (in the Table S3.A) are used to provide different scenarios of simulated isotopic value of consumer (δ_c). From δ_c different methods of static mixing model (SMM) are applied and the bias estimates ($\beta_{\frac{\omega}{\lambda}}$) allow to compare them. Before the use of the metric ω/λ a preliminary test is carried out to explore the respective effects of ω and λ on $\beta_{\frac{\omega}{\lambda}}$, using a bubble plot (see the first graph S3.B). The bubble plot is applied only on instantaneous static mixing model (SMM_t) with constant food sources (δ_s) over time. To explore also the impact of dynamic δ_s over time and the integrated static mixing model ($SMM_{\Delta t}$) method a pseudo-sensitivity analysis is conducted (in the second graph S3.C).

S3.A Table of collected data (ω, λ, $\beta_{\frac{\omega}{\lambda}}$) used to build the bubble plot to explore the independent effects of ω and λ on $\beta_{\frac{\omega}{\lambda}}$ estimates.

ω	λ	ω/λ	$\beta_{\frac{\omega}{\lambda}}$
0.002	0.002	1.00	63.3
0.004	0.002	2.00	54.9
0.008	0.002	4.00	53.4
0.010	0.002	5.00	56.2
0.002	0.020	0.10	10.1
0.004	0.020	0.20	19.8
0.008	0.020	0.40	34.6
0.010	0.020	0.50	39.2
0.002	0.100	0.02	2.1
0.004	0.100	0.04	4.0
0.008	0.100	0.08	8.0
0.010	0.100	0.10	10.3
0.002	0.200	0.01	1.1
0.004	0.200	0.02	2.0
0.008	0.200	0.04	4.0
0.010	0.200	0.05	5.3

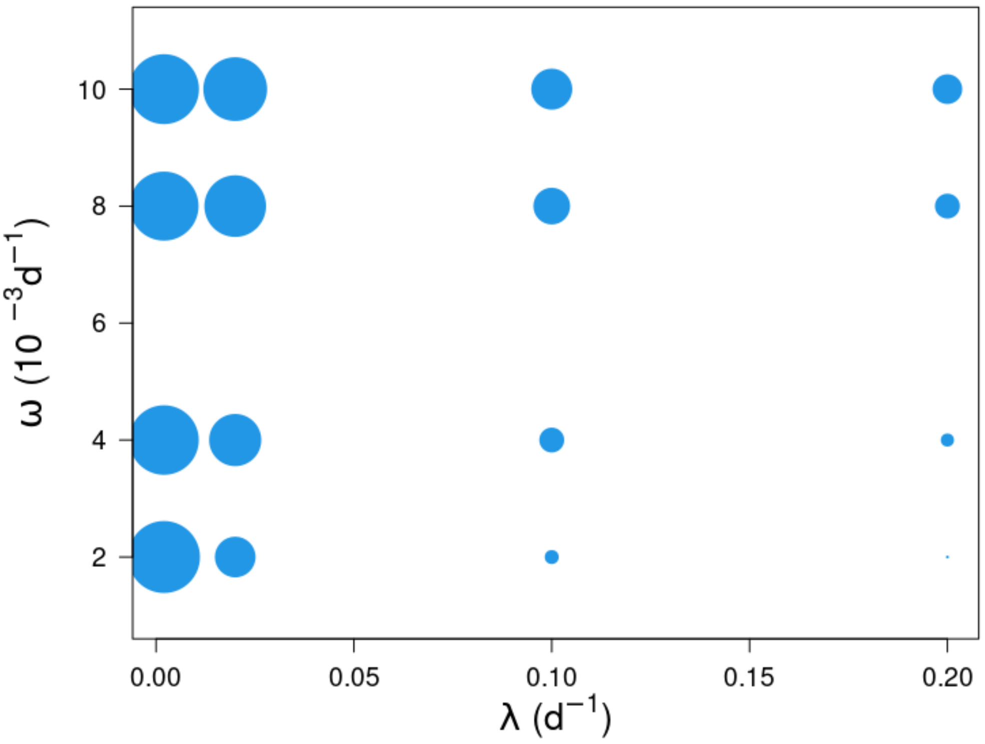

S3.B Bubble plot representing the bias ($\beta_{\frac{\omega}{\lambda}}$) of SMM_t application. The size of the bubble is proportional to the value of $\beta_{\frac{\omega}{\lambda}}$ estimates.

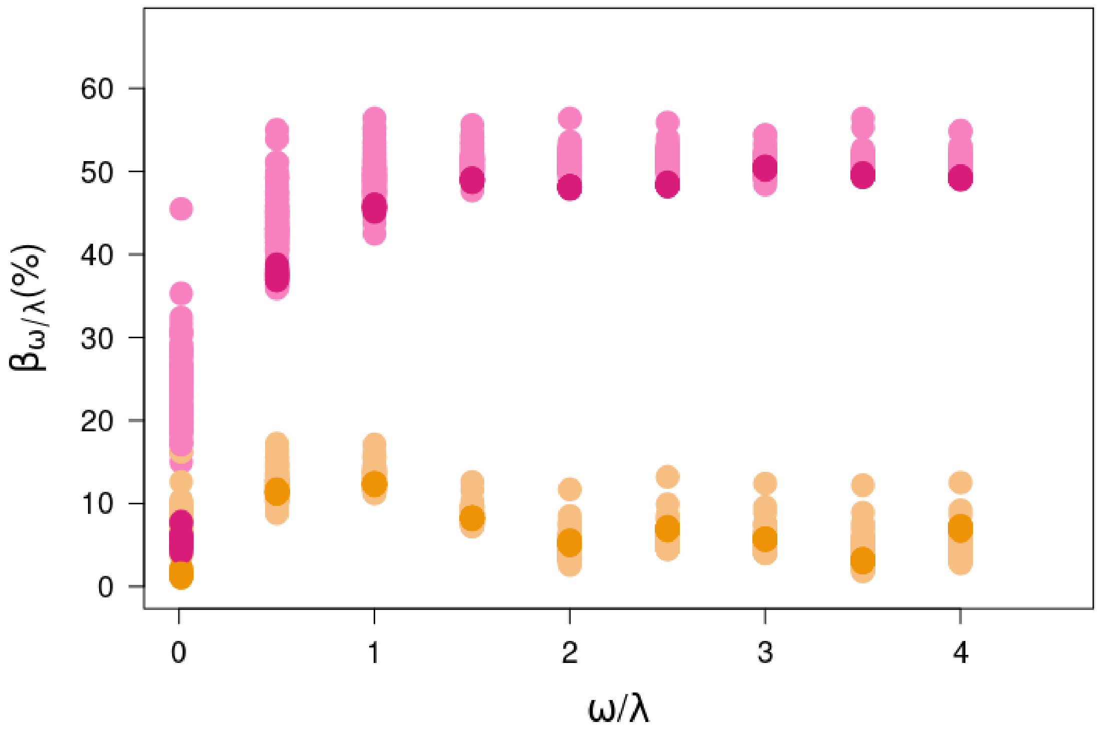

S3.C Pseudo-sensitivity analysis for $\beta_{\frac{\omega}{\lambda}}$ related to the ratio ω/λ – used as metric to explore the different SMM methods – with dynamic δ_s as Brownian trajectories and the two methods: $\mathrm{SMM_t}$ (pink colors) and $\mathrm{SMM_{\Delta t}}$ (orange colors) with $\Delta t = 2 \times t_{1/2}$. For simplicity and practicality, two λ values are used, $\lambda = 0.1$ d^{-1}(dark color) and $\lambda = 0.01$ d^{-1}(light color) for the 100 Brownian trajectories. Each point represent one scenario $\{\omega;\ \lambda;\ \delta_s\}$ and so one $\beta_{\frac{\omega}{\lambda}}$ estimation.

The variance of $\beta_{\frac{\omega}{\lambda}}$ for different combination of $\{\omega;\ \lambda\}$ is lower than the variance with Brownian sources for the same value of λ. Nevertheless, the variance is amplified by the coupling of low λ value and Brownian sources. The low variance induced by different combinations of ω, λ values – compared to that induced by the Brownian motion in food sources – justifies the choice of the metric ratio ω/λ.
